# Supplementary material for: Cardiac effects of OPA1 protein promotion in a transgenic animal model
Source: PLoS One. 2024 Nov 21;19(11):e0310394. doi: 10.1371/journal.pone.0310394 (PMC11581344; doi:10.1371/journal.pone.0310394)

Supplementary information for Figure 2.

WT	Positive control	TG
12,045	26,856	10,676
15,016	22,697	12,685
12,599	25,207	12,099
12,361	23,251	10,974
10,685	25,927	14,508
9,118	23,709	13,373
9,565	25,517	14,64
14,665	19,973	13,327
15,243	21,585	14,667
12,584	28,469	12,758
17,001	22,987	13,146
12,433	21,317	13,973
14,659	22,144	10,349
16,474	23,014	12,637
13,096	22,841	11,925
13,828	20,986	12,684
11,368	26,257	11,183
10,51	25,364	11,571

**MEAN**	12,95833333	23,78339	12,62083
**SD**	2,254767863	2,290282	1,341699
**Count**	18	18	18
**SEM**	0,531453882	0,539825	0,316242

**WT vs TG**	0,588808077
**WT vs Positive Control**	**6,18797E-16**
**TG vs Positive Control**	**7,77406E-19**

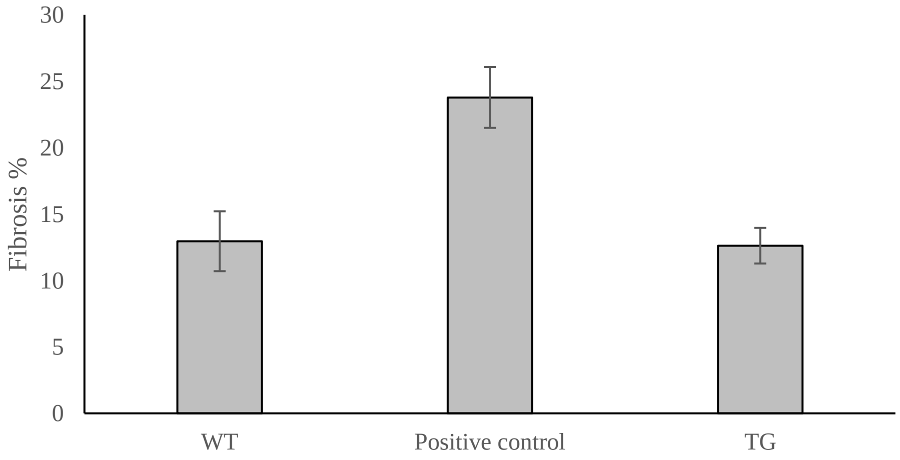

Supplement: S1 Fig — (PDF) [file pone.0310394.s001.pdf]
