# Supplementary figures and images for: Cardiac effects of OPA1 protein promotion in a transgenic animal model
Source: PLoS One. 2024 Nov 21;19(11):e0310394. doi: 10.1371/journal.pone.0310394 (PMC11581344; doi:10.1371/journal.pone.0310394)

Supplementary information for Figure 3.

| WT_3_4_2 | WT_7_7_2 | WT_7_7_4 | WT_8_2_2 | WT_10_1_4 | WT_11_1_2 | TG_3_4_1 | TG_4_5_2 | TG_7_7_3 | TG_8_2_1 | TG_10_1_1 | TG_10_1_2 |
|----------|----------|----------|----------|-----------|-----------|----------|----------|----------|----------|-----------|-----------|
| 27,739   | 20,914   | 23,13    | 14,385   | 19,751    | 21,284    | 17,391   | 17,464   | 21,098   | 25,878   | 23,093    | 23,438    |
| 22,106   | 22,815   | 25,861   | 14,779   | 17,918    | 20,494    | 18,596   | 20,175   | 19,8     | 28,022   | 20,661    | 19,038    |
| 21,01    | 17,337   | 22,507   | 15,497   | 19,26     | 17,58     | 19,825   | 16,37    | 20,948   | 20,232   | 22,824    | 16,995    |
| 23,238   | 20,388   | 25,97    | 18,537   | 14,746    | 14,308    | 18,835   | 21,804   | 24,994   | 24,239   | 18,108    | 20,555    |
| 20,225   | 23,287   | 16,181   | 15,455   | 13,286    | 14,984    | 19,336   | 14,558   | 25,346   | 18,619   | 18,877    | 20,499    |
| 24,565   | 19,058   | 19,709   | 14,43    | 15,914    | 21,866    | 20,891   | 16,608   | 24,638   | 18,507   | 26,812    | 19,009    |
| 21,075   | 17,076   | 23,436   | 15,455   | 19,977    | 15,716    | 18,493   | 19,41    | 23,882   | 22,971   | 16,979    | 19,098    |
| 19,372   | 19,424   | 26,965   | 16,103   | 15,089    | 20,59     | 20,271   | 18,554   | 21,426   | 21,305   | 19,105    | 17,936    |
| 19,922   | 17,447   | 20,73    | 19,047   | 15,466    | 18,814    | 19,468   | 24,652   | 21,337   | 20,975   | 17,971    | 19,363    |
| 16,099   | 21,958   | 22,79    | 18,502   | 19,848    | 16,733    | 16,292   | 17,656   | 18,716   | 22,075   | 15,533    | 15,678    |
| 25,482   | 23,478   | 17,972   | 14,518   | 19,293    | 18,602    | 18,665   | 18,218   | 25,221   | 17,699   | 16,611    | 20,856    |
| 16,957   | 17,702   | 16,225   | 14,866   | 18,48     | 16,968    | 15,936   | 16,507   | 22,992   | 24,095   | 19,17     | 13,588    |
| 15,238   | 21,036   | 19,996   | 18,979   | 17,152    | 21,222    | 16,135   | 16,642   | 17,458   | 19,603   | 15,089    | 14,938    |
| 17,773   | 19,058   | 24,879   | 18,742   | 15,954    | 18,495    | 17,464   | 12,349   | 19,927   | 22,85    | 15,888    | 13,998    |
| 16,015   | 19,358   | 20,384   | 18,364   | 16,583    | 16,733    | 17,897   | 15,345   | 25,718   | 18,731   | 21,627    | 13,754    |
| 18       | 19,783   | 19,123   | 15,079   | 19,16     | 16,253    | 18,97    | 17,751   | 21,602   | 23,392   | 23,2      | 19,392    |
| 21,329   | 19,621   | 20,384   | 19,738   | 17,411    | 16,374    | 16,793   | 12,303   | 18,063   | 22,201   | 24,598    | 19,538    |
| 19,197   | 19,325   | 23,497   | 17,252   | 17,738    | 16,654    | 19,728   | 15,884   | 31,104   | 23,746   | 17,088    | 18,403    |
| 17,544   | 19,058   | 18,518   | 15,414   | 22,788    | 19,537    | 18,25    | 18,033   | 22,687   | 18,088   | 22,688    | 18,434    |
| 18,663   | 19,125   | 19,086   | 19,771   | 19,193    | 26,582    | 17,718   | 14,975   | 18,512   | 22,232   | 24,548    | 17,904    |
| 20,391   | 15,547   | 21,604   | 16,261   | 16,034    | 17,277    | 16,448   | 17,656   | 25,246   | 31,507   | 22,247    | 12,593    |
| 16,225   | 14,702   | 21,471   | 20,748   | 19,293    | 21,097    | 19,921   | 19,697   | 23,019   | 25,959   | 13,982    | 17,062    |
| 15,545   | 21,783   | 20,523   | 21,144   | 19,719    | 20,75     | 19,889   | 18,584   | 12,319   | 27,823   | 16,574    | 12,948    |
| 12,405   | 18,514   | 19,491   | 16,649   | 18,857    | 19,162    | 31,346   | 19,439   | 19,543   | 20,301   | 15,927    | 17,904    |
| 15,675   | 23,038   | 27,358   | 14,341   | 16,034    | 26,706    | 19,985   | 21,958   | 23,829   | 19,281   | 20,329    | 14,593    |
| 16,837   | 21,665   | 17,893   | 16,063   | 14,746    | 18,989    | 19,104   | 18,614   | 19,478   | 21,041   | 18,58     | 18,061    |
| 11,443   | 20,103   | 22,601   | 16,417   | 18,515    | 22,985    | 24,149   | 14,048   | 15,045   | 24,779   | 22,524    | 13,546    |
| 15,846   | 16,073   | 26,324   | 16,687   | 16,074    | 17,655    | 19,204   | 18,764   | 17,055   | 23,001   | 13,209    | 18,617    |
| 13,042   | 17,665   | 18,974   | 20,091   | 19,326    | 19,058    | 15,24    | 17,687   | 15,378   | 22,388   | 12,585    | 15,714    |
| 16,39    | 14,658   | 21,338   | 22,377   | 23,343    | 15,631    | 26,031   | 17,939   | 12,92    | 19,099   | 17,693    | 16,211    |
| 20,752   | 16,427   | 20,488   | 18,364   | 18,06     | 16,293    | 22,985   | 21,65    | 15,129   | 20,059   | 16,12     | 12,991    |
| 19,477   | 17,556   | 22,696   | 16,726   | 18,891    | 19,265    | 17,28    | 15,013   | 16,374   | 24,468   | 14,114    | 17,455    |
| 20,091   | 18,024   | 18,364   | 15,742   | 20,326    | 16,851    | 22,79    | 17,205   | 18,306   | 18,356   | 14,631    | 16,726    |
| 18,444   | 17,076   | 22,124   | 18,912   | 13,712    | 27,22     | 25,108   | 17,238   | 18,306   | 19,92    | 19,395    | 16,895    |
| 20,49    | 14,658   | 19,745   | 16,878   | 15,793    | 21,592    | 16,331   | 16,943   | 14,046   | 18,989   | 17,376    | 16,07     |
| 18,914   | 17,953   | 18,595   | 15,701   | 17,846    | 17,353    | 15,855   | 23,631   | 15,46    | 18,242   | 18,713    | 16,211    |
| 22,5     | 18,13    | 21,205   | 13,888   | 15,089    | 20,005    | 20,614   | 20,396   | 16,868   | 20,471   | 18,176    | 17,967    |
| 22,198   | 18,72    | 20,208   | 17,509   | 19,491    | 16,733    | 26,946   | 21,125   | 19,575   | 25,905   | 16,158    | 19,363    |
| 23,44    | 13,189   | 20,102   | 17,87    | 14,035    | 19,905    | 26,589   | 19,207   | 19,216   | 25,224   | 21,193    | 13,207    |
| 16,266   | 17,52    | 17,286   | 17,252   | 15,507    | 19,772    | 25,082   | 22,212   | 19,018   | 20,842   | 17,411    | 16,76     |
| 17,962   | 20,606   | 15,507   | 15,823   | 13,897    | 18,28     | 22,929   | 19,119   | 14,001   | 28,097   | 17,623    | 18,465    |
| 16,917   | 19,259   | 13,757   | 16,953   | 17,953    | 21,408    | 24,929   | 23,843   | 14,401   | 24,975   | 15,77     | 16,659    |
| 16,595   | 13,897   | 24,936   | 20,624   | 18,166    | 12,589    | 20,891   | 17,845   | 13,066   | 23,775   | 15,965    | 16,07     |
| 12,886   | 14,034   | 16,743   | 16,764   | 19,193    | 23,27     | 19,204   | 22,563   | 14,919   | 24,153   | 17,411    | 18,678    |
| 15,675   | 22,759   | 11,504   | 16,953   | 17,882    | 12,378    | 21,552   | 16,876   | 14,876   | 17,422   | 18,346    | 16,625    |
| 12,833   | 15,506   | 20,243   | 25,398   | 18,341    | 22,016    | 19,171   | 14,9     | 13,955   | 24,182   | 18,108    | 24,246    |
| 15,282   | 23,259   | 16,312   | 21,355   | 19,784    | 18,602    | 23,561   | 19,265   | 20,022   | 23,54    | 18,943    | 17,062    |
| 17,233   | 16,812   | 14,899   | 20,344   | 15,914    | 17,767    | 28,845   | 19,951   | 17,638   | 23,805   | 15,007    | 22,752    |
| 16,266   | 19,621   | 13,548   | 15,497   | 15,548    | 20,104    | 16,869   | 23,702   | 17,276   | 19,815   | 13,255    | 23,244    |
| 16,349   | 21,871   | 17,572   | 19,18    | 18,992    | 16,495    | 19,071   | 21,494   | 17,53    | 25,28    | 18,21     | 22,375    |
| 13,797   | 18,095   | 14,215   | 16,953   | 16,544    | 20,136    | 16,869   | 18,824   | 15,254   | 21,821   | 20,721    | 17,777    |
| 13,894   | 22,391   | 20,935   | 14,296   | 21,067    | 16,694    | 19,599   | 18,187   | 15,943   | 19,744   | 21,939    | 24,409    |
| 11,906   | 28,023   | 16,004   | 18,946   | 17,374    | 19,739    | 18,528   | 17,107   | 19,051   | 14,925   | 16,943    | 15,929    |
| 16,183   | 19,058   | 17,893   | 15,497   | 15,793    | 15,246    | 16,015   | 19,002   | 14,834   | 22,758   | 22,024    | 17,553    |
| 16,141   | 21,871   | 21,833   | 14,736   | 17,702    | 20,038    | 17,646   | 21,701   | 15,784   | 19,567   | 18,975    | 20,747    |
| 16,877   | 19,325   | 24,793   | 17,326   | 18,024    | 17,046    | 19,664   | 17,656   | 19,117   | 20,842   | 17,411    | 20,774    |
| 16,472   | 18,856   | 15,78    | 15,944   | 17,953    | 15,924    | 19,27    | 19,41    | 17,166   | 20,573   | 18,58     | 13,754    |
| 15,015   | 19,192   | 19,455   | 12,787   | 15,215    | 18,849    | 19,857   | 17,908   | 16,868   | 20,301   | 17,763    | 23,535    |
| 20,457   | 19,391   | 20,243   | 19,771   | 15,752    | 16,048    | 23,398   | 21,65    | 18,063   | 18,507   | 18,04     | 17,999    |
| 21,234   | 16,073   | 17,572   | 21,295   | 18,131    | 18,884    | 24,464   | 22,612   | 20,117   | 19,815   | 23,883    | 19,509    |

|        |        |        |        |        |        |        |        |        |        |        |        |
|--------|--------|--------|--------|--------|--------|--------|--------|--------|--------|--------|--------|
| 18     | 15,993 | 16,356 | 16,991 | 16,467 | 18,673 | 18,145 | 21,441 | 21,396 | 19,317 | 24,447 | 16,726 |
| 17,697 | 19,092 | 18,671 | 17,691 | 15,257 | 14,215 | 22,48  | 18,523 | 22,037 | 22,543 | 18,142 | 20,499 |
| 21,953 | 17,988 | 19,637 | 17,87  | 22,363 | 17,391 | 15,365 | 16,709 | 15,46  | 19,744 | 16,759 | 15,275 |
| 15,06  | 19,912 | 17,079 | 19,313 | 15,131 | 18,779 | 19,857 | 16,709 | 17,458 | 17,017 | 19,267 | 20,305 |
| 19,162 | 15,382 | 18,936 | 20,281 | 28,544 | 22,166 | 18,936 | 15,454 | 21,719 | 20,471 | 16,272 | 23,122 |
| 16,877 | 18,059 | 20,661 | 17,326 | 24,804 | 12,378 | 19,171 | 17,107 | 16,451 | 18,356 | 22,024 | 14,437 |
| 15,501 | 19,292 | 23,284 | 20,84  | 19,589 | 21,222 | 19,204 | 17,592 | 15,663 | 17,778 | 18,142 | 15,605 |
| 21,55  | 19,588 | 26,025 | 16,378 | 18,686 | 17,878 | 19,728 | 20,478 | 15,663 | 21,435 | 22,524 | 17,649 |
| 21,83  | 11,807 | 17,409 | 21,235 | 19,425 | 12,324 | 14,334 | 17,782 | 18,202 | 19,99  | 19,68  | 16,659 |
| 18,149 | 19,225 | 16,399 | 21,681 | 17,374 | 15,924 | 17,132 | 19,352 | 16,179 | 17,541 | 17,763 | 18,218 |
| 24,51  | 16,192 | 15,415 | 17,691 | 16,428 | 19,058 | 14,986 | 11,886 | 16,943 | 14,69  | 16,348 | 18,372 |
| 14,277 | 16,812 | 20,764 | 17,436 | 20,884 | 15,716 | 19,501 | 13,928 | 18,547 | 21,693 | 17,867 | 27,61  |
| 21,329 | 21,841 | 22,252 | 17,252 | 24,958 | 17,467 | 18,631 | 15,848 | 18,716 | 18,507 | 24,396 | 19,624 |
| 22,679 | 16,812 | 22,633 | 17,326 | 20,041 | 19,939 | 18,355 | 14,825 | 20,148 | 26,305 | 24,218 | 17,488 |
| 17,849 | 18,059 | 22,22  | 18,224 | 18,789 | 14,537 | 17,243 | 15,382 | 18,168 | 18,203 | 17,34  | 19,246 |
| 19,268 | 23,011 | 17,893 | 15,121 | 21,607 | 21,439 | 18,801 | 14,246 | 15,296 | 21,141 | 19,427 | 22,274 |
| 15,846 | 18,13  | 20,173 | 18,398 | 17,989 | 13,303 | 22,565 | 16,976 | 17,055 | 19,638 | 22,275 | 16,035 |
| 16,957 | 21,428 | 22,188 | 17,066 | 20,638 | 15,674 | 18,97  | 17,238 | 19,183 | 15,248 | 22,606 | 16,105 |
| 18,914 | 21,871 | 18,633 | 22,09  | 20,357 | 17,618 | 24,646 | 19,923 | 21,038 | 23,092 | 19,585 | 10,536 |
| 16,877 | 16,388 | 15,825 | 16,222 | 17,484 | 13,791 | 20,891 | 15,813 | 13,448 | 23,717 | 23,227 | 17,292 |
| 15,238 | 16,466 | 14,946 | 21,533 | 20,168 | 18,28  | 20,396 | 15,884 | 14,919 | 18,544 | 20,115 | 15,46  |
| 18,481 | 14,034 | 13,548 | 20,779 | 20,483 | 18,849 | 19,825 | 17,107 | 19,959 | 17,778 | 20,601 | 20,165 |
| 13,649 | 23,776 | 13,601 | 16,533 | 24,283 | 15,758 | 22,309 | 17,107 | 17,018 | 17,895 | 23,28  | 18,311 |
| 18,95  | 14,571 | 11,565 | 14,206 | 20,514 | 14,075 | 18,493 | 17,751 | 16,101 | 13,961 | 20,988 | 9,754  |
| 17,582 | 18,375 | 16,486 | 20,059 | 19,359 | 20,104 | 15,156 | 14,008 | 22,575 | 17,18  | 19,458 | 16     |
| 17,389 | 13,524 | 16,137 | 16,103 | 22,075 | 13,886 | 20,459 | 20,341 | 18,512 | 20,301 | 20,661 | 20,444 |
| 13,991 | 22,276 | 12,454 | 19,247 | 15,954 | 24,487 | 18,459 | 16,943 | 17,166 | 18,989 | 14,673 | 20,91  |
| 18     | 17,917 | 12,281 | 20,993 | 21,872 | 17,655 | 17,646 | 19,294 | 16,755 | 19,885 | 16,574 | 19,275 |
| 19,162 | 15,953 | 16,004 | 15,863 | 18,236 | 19,401 | 18,389 | 15,563 | 18,581 | 19,99  | 16,906 | 18,093 |
| 23,15  | 16,888 | 13,601 | 21,769 | 17,374 | 17,58  | 17,243 | 15,527 | 17,566 | 15,742 | 19,617 | 17,26  |
| 18,735 | 15,34  | 17,612 | 17,941 | 23,858 | 16,212 | 21,194 | 12,027 | 18,884 | 19,674 | 11,669 | 18,186 |
| 18,699 | 16,466 | 16,7   | 15,661 | 22,788 | 16,253 | 17,933 | 17,624 | 17,494 | 20,775 | 20,781 | 17,161 |
| 20,091 | 15,173 | 18,861 | 23,245 | 22,928 | 20,622 | 21,992 | 17,464 | 18,133 | 18,768 | 24,115 | 10,536 |
| 20,654 | 21,812 | 17,037 | 14,341 | 20,389 | 18,884 | 17,02  | 17,496 | 19,117 | 22,388 | 19,774 | 18,311 |
| 17,735 | 17,447 | 16,743 | 19,18  | 20,231 | 15,966 | 18,25  | 16,876 | 13,773 | 20,505 | 21,939 | 18,218 |
| 27     | 17,373 | 19,673 | 19,867 | 15,004 | 16,812 | 15,114 | 15,05  | 13,541 | 19,281 | 18,58  | 19,009 |
| 21,799 | 14,26  | 14,851 | 19,28  | 18,721 | 22,725 | 20,768 | 18,584 | 16,943 | 18,731 | 19,458 | 14,747 |
| 20,556 | 21,066 | 17,079 | 17,546 | 19,293 | 20,202 | 17,682 | 15,124 | 15,704 | 16,687 | 21,655 | 17,488 |
| 20,391 | 15,589 | 22,348 | 19,706 | 24,071 | 18,779 | 16,331 | 14,128 | 18,918 | 19,46  | 19,899 | 12,366 |
| 25,745 | 15,215 | 21,002 | 19,899 | 16,232 | 18,673 | 17,464 | 14,008 | 23,265 | 25,086 | 18,48  | 14,437 |

|          |          |          |          |          |          |          |          |          |          |          |          |
|----------|----------|----------|----------|----------|----------|----------|----------|----------|----------|----------|----------|
| 18,39176 | 18,50551 | 19,14459 | 17,81158 | 18,55571 | 18,30296 | 19,61247 | 17,82997 | 18,45174 | 20,95058 | 19,03856 | 17,70678 |
|----------|----------|----------|----------|----------|----------|----------|----------|----------|----------|----------|----------|

|         |           |           |
|---------|-----------|-----------|
|         | WT        | TG        |
| mean    | 18,452018 | 18,931683 |
| SD      | 0,4311101 | 1,2243646 |
| n       | 6         | 6         |
| SEM     | 0,176     | 0,4998447 |
| t probe | 0,386671  |           |

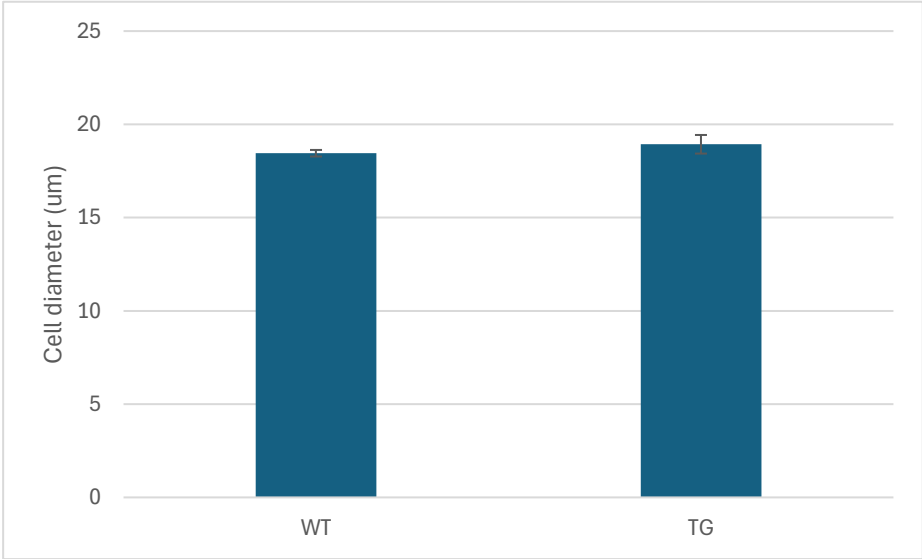

Supplement: S2 Fig — (PDF) [file pone.0310394.s002.pdf]
