# Supplementary material for: Cardiac effects of OPA1 protein promotion in a transgenic animal model
Source: PLoS One. 2024 Nov 21;19(11):e0310394. doi: 10.1371/journal.pone.0310394 (PMC11581344; doi:10.1371/journal.pone.0310394)
Supplement: S3 Fig — (PDF) [file pone.0310394.s003.pdf]

Supplementary information for Figure 4.

8-oxoguanin

	Label	Area	Mean	%Area	MinThr	MaxThr
1	OT11.20.0x	55804	181,592	8,081	150	200
2	OT12.20.0x	44387	180,314	6,427	150	200
3	OT13.20.0x	59325	181,467	8,59	150	200
4	OT21.20.0x	51292	180,15	7,427	150	200
5	OT22.20.0x	35746	179,078	5,176	150	200
6	OT23.20.0x	52895	180,386	7,659	150	200
7	OT31.20.0x	54206	180,009	7,849	150	200
8	OT32.20.0x	58494	181,012	8,47	150	200
9	OT33.tif:Bl	74769	181,534	10,827	150	200
10	OT41.tif:Bl	32653	179,069	4,728	150	200
11	OT42.tif:Bl	32254	179,177	4,67	150	200
12	OT43.tif:Bl	34828	179,957	5,043	150	200
13	OT51.tif:Bl	55152	180,743	7,986	150	200
14	OT52.tif:Bl	32245	178,468	4,669	150	200
15	OT53.tif:Bl	34079	177,489	4,935	150	200
16	OT61.tif:Bl	25100	177,452	3,635	150	200
17	OT62.tif:Bl	33385	177,758	4,834	150	200
18	OT63.tif:Bl	37126	180,346	5,376	150	200
19	OW11.tif:B	56589	181,252	8,194	150	200
20	OW12.tif:B	56467	180,623	8,177	150	200
21	OW13.tif:B	59072	180,064	8,554	150	200
22	OW21.tif:B	46821	179,142	6,78	150	200
23	OW22.tif:B	50073	181,618	7,251	150	200
24	OW23.tif:B	45373	181,352	6,57	150	200
25	OW31.tif:B	27769	177,57	4,021	150	200
26	OW32.tif:B	31625	178,756	4,579	150	200
27	OW33.tif:B	34518	181,299	4,998	150	200
28	OW41.tif:B	39869	178,467	5,773	150	200
29	OW42.tif:B	35456	178,098	5,134	150	200
30	OW43.tif:B	35537	178,45	5,146	150	200
31	OW51.tif:B	61827	177,375	8,953	150	200
32	OW52.tif:B	39938	177,133	5,783	150	200
33	OW53.tif:B	53156	178,316	7,697	150	200
34	OW61.tif:B	51178	178,412	7,411	150	200
35	OW62.tif:B	55232	178,17	7,998	150	200
36	OW63.tif:B	55603	178,206	8,051	150	200

	**WT**	**TG**
	8,194	8,081
	8,177	6,427
	8,554	8,59
	6,78	7,427
	7,251	5,176
	6,57	7,659
	4,021	7,849
	4,579	8,47
	4,998	10,827
	5,773	4,728
	5,134	4,67
	5,146	5,043
	8,953	7,986
	5,783	4,669
	7,697	4,935
	7,411	3,635
	7,998	4,834
	8,051	5,376
Mean	6,726111	6,465667
SD	1,522209	1,941798
SEM	0,358788	0,457686
n	18	18
t probe	0,657108	

Nitrotyrosin

	Label	Area	Mean	%Area	MinThr	MaxThr
1	TG11.tif:Bl	116232	183,428	16,831	150	200
2	TG12.tif:Bl	60495	182,716	8,76	150	200
3	TG13.tif:Bl	176223	186,367	25,517	150	200
4	TG21.tif:Bl	68393	183,421	9,903	150	200
5	TG22.tif:Bl	142480	183,949	20,631	150	200
6	TG23.tif:Bl	77250	184,265	11,186	150	200
7	TG31.tif:Bl	51407	181,784	7,444	150	200
8	TG32.tif:Bl	97086	185,656	14,058	150	200
9	TG33.tif:Bl	164982	184,4	23,89	150	200
10	TG41.tif:Bl	100514	183,54	14,555	150	200
11	TG42.tif:Bl	138046	182,699	19,989	150	200
12	TG43.tif:Bl	99788	183,954	14,449	150	200
13	TG51.tif:Bl	115471	186,578	16,72	150	200
14	TG52.tif:Bl	198672	187,274	28,768	150	200
15	TG53.tif:Bl	110006	185,855	15,929	150	200
16	TG61.tif:Re	115958	187,644	16,791	150	200
17	TG62.tif:Bl	134292	183,503	19,446	150	200
18	TG63.tif:Bl	65962	183,857	9,551	150	200
19	WT11.tif:B	218297	184,348	31,61	150	200
20	WT12.tif:B	215627	185,364	31,223	150	200
21	WT13.tif:B	127726	184,279	18,495	150	200
22	WT21.tif:B	351418	182,816	50,886	150	200
23	WT23.tif:B	87750	185,358	12,706	150	200
24	WT22.tif:B	61822	184,776	8,952	150	200
25	WT31.tif:B	81091	184,9	11,742	150	200
26	WT32.tif:B	94079	183,795	13,623	150	200
27	WT33.tif:B	198162	185,284	28,694	150	200
28	WT41.tif:B	120865	185,652	17,501	150	200
29	WT42.tif:B	162611	185,688	23,546	150	200
30	WT43.tif:B	66350	183,37	9,608	150	200
31	WT51.tif:B	57551	184,065	8,333	150	200
32	WT52.tif:B	90372	185,608	13,086	150	200
33	WT53.tif:B	97636	185,751	14,138	150	200
34	WT61.tif:B	51706	183,321	7,487	150	200
35	WT62.tif:B	63333	183,251	9,171	150	200
36	WT63.tif:B	67044	184,6	9,708	150	200

	**WT**	**TG**
	31,61	16,831
	31,223	8,76
	18,495	25,517
	50,886	9,903
	12,706	20,631
	8,952	11,186
	11,742	7,444
	13,623	14,058
	28,694	23,89
	17,501	14,555
	23,546	19,989
	9,608	14,449
	8,333	16,72
	13,086	28,768
	14,138	15,929
	7,487	16,791
	9,171	19,446
	9,708	9,551
Mean	17,80606	16,35656
SD	11,41436	5,943148
SEM	2,690391	1,400813
n	18	18
t probe	0,635798	
