# Supplementary material for: Cardiac effects of OPA1 protein promotion in a transgenic animal model
Source: PLoS One. 2024 Nov 21;19(11):e0310394. doi: 10.1371/journal.pone.0310394 (PMC11581344; doi:10.1371/journal.pone.0310394)
Supplement: S4 Fig — (PDF) [file pone.0310394.s004.pdf]

Supplementary information for Figure 5.

**WT**

| | Label | Area |
|---|---|---|
| 1 | Guest_IMG | 0,421 |
| 2 | Guest_IMG | 0,884 |
| 3 | Guest_IMG | 0,43 |
| 4 | Guest_IMG | 0,51 |
| 5 | Guest_IMG | 0,734 |
| 6 | Guest_IMG | 0,595 |
| 7 | Guest_IMG | 0,727 |
| 8 | Guest_IMG | 0,986 |
| 9 | Guest_IMG | 0,539 |
| 10 | Guest_IMG | 0,327 |
| 11 | Guest_IMG | 0,424 |
| 12 | Guest_IMG | 0,24 |
| 13 | Guest_IMG | 0,171 |
| 14 | Guest_IMG | 0,281 |
| 15 | Guest_IMG | 0,885 |
| 16 | Guest_IMG | 0,398 |
| 17 | Guest_IMG | 0,618 |
| 18 | Guest_IMG | 0,571 |
| 19 | Guest_IMG | 0,387 |
| 20 | Guest_IMG | 0,341 |
| 21 | Guest_IMG | 0,49 |
| 22 | Guest_IMG | 0,465 |
| 23 | Guest_IMG | 0,639 |
| 24 | Guest_IMG | 0,974 |
| 25 | Guest_IMG | 0,489 |
| 26 | Guest_IMG | 0,463 |
| 27 | Guest_IMG | 0,679 |
| 28 | Guest_IMG | 0,442 |
| 29 | Guest_IMG | 0,363 |
| 30 | Guest_IMG | 0,295 |
| 31 | Guest_IMG | 0,232 |
| 32 | Guest_IMG | 0,701 |
| 33 | Guest_IMG | 0,446 |
| 34 | Guest_IMG | 0,412 |
| 35 | Guest_IMG | 0,62 |
| 36 | Guest_IMG | 0,368 |
| 37 | Guest_IMG | 0,441 |
| 38 | Guest_IMG | 0,238 |
| 39 | Guest_IMG | 0,923 |
| 40 | Guest_IMG | 0,266 |
| 41 | Guest_IMG | 0,136 |
| 42 | Guest_IMG | 1,122 |
| 43 | Guest_IMG | 0,375 |
| 44 | Guest_IMG | 0,59 |
| 45 | Guest_IMG | 1,382 |

**TG**

| | Label | Area |
|---|---|---|
| 1 | Guest_IMG | 0,828 |
| 2 | Guest_IMG | 0,9 |
| 3 | Guest_IMG | 1,631 |
| 4 | Guest_IMG | 0,104 |
| 5 | Guest_IMG | 0,235 |
| 6 | Guest_IMG | 0,262 |
| 7 | Guest_IMG | 0,714 |
| 8 | Guest_IMG | 0,897 |
| 9 | Guest_IMG | 0,583 |
| 10 | Guest_IMG | 0,666 |
| 11 | Guest_IMG | 0,548 |
| 12 | Guest_IMG | 0,251 |
| 13 | Guest_IMG | 2,5 |
| 14 | Guest_IMG | 0,257 |
| 15 | Guest_IMG | 0,821 |
| 16 | Guest_IMG | 0,518 |
| 17 | Guest_IMG | 0,272 |
| 18 | Guest_IMG | 0,805 |
| 19 | Guest_IMG | 0,504 |
| 20 | Guest_IMG | 1,266 |
| 21 | Guest_IMG | 0,884 |
| 22 | Guest_IMG | 0,698 |
| 23 | Guest_IMG | 0,529 |
| 24 | Guest_IMG | 0,521 |
| 25 | Guest_IMG | 0,325 |
| 26 | Guest_IMG | 0,732 |
| 27 | Guest_IMG | 0,228 |
| 28 | Guest_IMG | 0,175 |
| 29 | Guest_IMG | 0,772 |
| 30 | Guest_IMG | 0,532 |
| 31 | Guest_IMG | 0,593 |
| 32 | Guest_IMG | 0,535 |
| 33 | Guest_IMG | 0,341 |
| 34 | Guest_IMG | 0,239 |
| 35 | Guest_IMG | 0,489 |
| 36 | Guest_IMG | 0,639 |
| 37 | Guest_IMG | 0,298 |
| 38 | Guest_IMG | 0,793 |
| 39 | Guest_IMG | 0,543 |
| 40 | Guest_IMG | 0,694 |
| 41 | Guest_IMG | 0,373 |
| 42 | Guest_IMG | 0,926 |
| 43 | Guest_IMG | 0,439 |
| 44 | Guest_IMG | 0,374 |
| 45 | Guest_IMG | 0,248 |

| WT | TG |
|---|---|
| 0,421 | 0,828 |
| 0,884 | 0,9 |
| 0,43 | 1,631 |
| 0,51 | 0,104 |
| 0,734 | 0,235 |
| 0,595 | 0,262 |
| 0,727 | 0,714 |
| 0,986 | 0,897 |
| 0,539 | 0,583 |
| 0,327 | 0,666 |
| 0,424 | 0,548 |
| 0,24 | 0,251 |
| 0,171 | 2,5 |
| 0,281 | 0,257 |
| 0,885 | 0,821 |
| 0,398 | 0,518 |
| 0,618 | 0,272 |
| 0,571 | 0,805 |
| 0,387 | 0,504 |
| 0,341 | 1,266 |
| 0,49 | 0,884 |
| 0,465 | 0,698 |
| 0,639 | 0,529 |
| 0,974 | 0,521 |
| 0,489 | 0,325 |
| 0,463 | 0,732 |
| 0,679 | 0,228 |
| 0,442 | 0,175 |
| 0,363 | 0,772 |
| 0,295 | 0,532 |
| 0,232 | 0,593 |
| 0,701 | 0,535 |
| 0,446 | 0,341 |
| 0,412 | 0,239 |
| 0,62 | 0,489 |
| 0,368 | 0,639 |
| 0,441 | 0,298 |
| 0,238 | 0,793 |
| 0,923 | 0,543 |
| 0,266 | 0,694 |
| 0,136 | 0,373 |
| 1,122 | 0,926 |
| 0,375 | 0,439 |
| 0,59 | 0,374 |
| 1,382 | 0,248 |

| | | |
|---|---|---|
| 46 | Guest_IMG | 0,296 |
| 47 | Guest_IMG | 0,495 |
| 48 | Guest_IMG | 0,567 |
| 49 | Guest_IMG | 0,497 |
| 50 | Guest_IMG | 0,469 |
| 51 | Guest_IMG | 0,745 |
| 52 | Guest_IMG | 0,638 |
| 53 | Guest_IMG | 0,638 |
| 54 | Guest_IMG | 1,312 |
| 55 | Guest_IMG | 0,696 |
| 56 | Guest_IMG | 0,796 |
| 57 | Guest_IMG | 0,582 |
| 58 | Guest_IMG | 0,412 |
| 59 | Guest_IMG | 0,638 |
| 60 | Guest_IMG | 0,764 |
| 61 | Guest_IMG | 1,395 |
| 62 | Guest_IMG | 0,333 |
| 63 | Guest_IMG | 0,404 |
| 64 | Guest_IMG | 0,497 |
| 65 | Guest_IMG | 0,276 |
| 66 | Guest_IMG | 0,746 |
| 67 | Guest_IMG | 0,22 |
| 68 | Guest_IMG | 0,68 |
| 69 | Guest_IMG | 0,567 |
| 70 | Guest_IMG | 1,861 |
| 71 | Guest_IMG | 0,563 |
| 72 | Guest_IMG | 0,595 |
| 73 | Guest_IMG | 0,56 |
| 74 | Guest_IMG | 0,615 |
| 75 | Guest_IMG | 0,28 |
| 76 | Guest_IMG | 0,308 |
| 77 | Guest_IMG | 0,589 |
| 78 | Guest_IMG | 0,474 |
| 79 | Guest_IMG | 0,421 |
| 80 | Guest_IMG | 0,539 |
| 81 | Guest_IMG | 0,601 |
| 82 | Guest_IMG | 0,338 |
| 83 | Guest_IMG | 0,691 |
| 84 | Guest_IMG | 0,395 |
| 85 | Guest_IMG | 0,976 |
| 86 | Guest_IMG | 0,583 |
| 87 | Guest_IMG | 0,67 |
| 88 | Guest_IMG | 0,876 |
| 89 | Guest_IMG | 0,509 |
| 90 | Guest_IMG | 0,495 |
| 91 | Guest_IMG | 0,667 |
| 92 | Guest_IMG | 0,699 |
| 93 | Guest_IMG | 0,619 |
| 94 | Guest_IMG | 0,378 |
| 95 | Guest_IMG | 0,496 |

| | | |
|---|---|---|
| 46 | Guest_IMG | 0,448 |
| 47 | Guest_IMG | 0,248 |
| 48 | Guest_IMG | 0,261 |
| 49 | Guest_IMG | 0,297 |
| 50 | Guest_IMG | 0,189 |
| 51 | Guest_IMG | 0,514 |
| 52 | Guest_IMG | 0,341 |
| 53 | Guest_IMG | 0,389 |
| 54 | Guest_IMG | 0,573 |
| 55 | Guest_IMG | 1,037 |
| 56 | Guest_IMG | 0,847 |
| 57 | Guest_IMG | 0,316 |
| 58 | Guest_IMG | 0,474 |
| 59 | Guest_IMG | 0,827 |
| 60 | Guest_IMG | 0,846 |
| 61 | Guest_IMG | 0,821 |
| 62 | Guest_IMG | 0,554 |
| 63 | Guest_IMG | 0,761 |
| 64 | Guest_IMG | 0,394 |
| 65 | Guest_IMG | 0,514 |
| 66 | Guest_IMG | 0,76 |
| 67 | Guest_IMG | 0,548 |
| 68 | Guest_IMG | 0,606 |
| 69 | Guest_IMG | 0,807 |
| 70 | Guest_IMG | 0,531 |
| 71 | Guest_IMG | 0,47 |
| 72 | Guest_IMG | 0,366 |
| 73 | Guest_IMG | 0,371 |
| 74 | Guest_IMG | 0,558 |
| 75 | Guest_IMG | 0,342 |
| 76 | Guest_IMG | 0,712 |
| 77 | Guest_IMG | 0,476 |
| 78 | Guest_IMG | 0,785 |
| 79 | Guest_IMG | 0,557 |
| 80 | Guest_IMG | 0,469 |
| 81 | Guest_IMG | 0,311 |
| 82 | Guest_IMG | 0,486 |
| 83 | Guest_IMG | 0,663 |
| 84 | Guest_IMG | 0,782 |
| 85 | Guest_IMG | 0,512 |
| 86 | Guest_IMG | 0,459 |
| 87 | Guest_IMG | 1,199 |
| 88 | Guest_IMG | 0,324 |
| 89 | Guest_IMG | 0,65 |
| 90 | Guest_IMG | 0,43 |
| 91 | Guest_IMG | 0,133 |
| 92 | Guest_IMG | 0,446 |
| 93 | Guest_IMG | 1,601 |
| 94 | Guest_IMG | 0,863 |
| 95 | Guest_IMG | 0,163 |

| | |
|---|---|
| 0,296 | 0,448 |
| 0,495 | 0,248 |
| 0,567 | 0,261 |
| 0,497 | 0,297 |
| 0,469 | 0,189 |
| 0,745 | 0,514 |
| 0,638 | 0,341 |
| 0,638 | 0,389 |
| 1,312 | 0,573 |
| 0,696 | 1,037 |
| 0,796 | 0,847 |
| 0,582 | 0,316 |
| 0,412 | 0,474 |
| 0,638 | 0,827 |
| 0,764 | 0,846 |
| 1,395 | 0,821 |
| 0,333 | 0,554 |
| 0,404 | 0,761 |
| 0,497 | 0,394 |
| 0,276 | 0,514 |
| 0,746 | 0,76 |
| 0,22 | 0,548 |
| 0,68 | 0,606 |
| 0,567 | 0,807 |
| 1,861 | 0,531 |
| 0,563 | 0,47 |
| 0,595 | 0,366 |
| 0,56 | 0,371 |
| 0,615 | 0,558 |
| 0,28 | 0,342 |
| 0,308 | 0,712 |
| 0,589 | 0,476 |
| 0,474 | 0,785 |
| 0,421 | 0,557 |
| 0,539 | 0,469 |
| 0,601 | 0,311 |
| 0,338 | 0,486 |
| 0,691 | 0,663 |
| 0,395 | 0,782 |
| 0,976 | 0,512 |
| 0,583 | 0,459 |
| 0,67 | 1,199 |
| 0,876 | 0,324 |
| 0,509 | 0,65 |
| 0,495 | 0,43 |
| 0,667 | 0,133 |
| 0,699 | 0,446 |
| 0,619 | 1,601 |
| 0,378 | 0,863 |
| 0,496 | 0,163 |

| | | | | | | | |
|---|---|---|---|---|---|---|---|
| 96 | Guest_IMG | 0,319 | 96 | Guest_IMG | 0,767 | 0,319 | 0,767 |
| 97 | Guest_IMG | 0,487 | 97 | Guest_IMG | 0,568 | 0,487 | 0,568 |
| 98 | Guest_IMG | 0,463 | 98 | Guest_IMG | 0,586 | 0,463 | 0,586 |
| 99 | Guest_IMG | 0,457 | 99 | Guest_IMG | 0,271 | 0,457 | 0,271 |
| 100 | Guest_IMG | 0,603 | 100 | Guest_IMG | 0,446 | 0,603 | 0,446 |
| 101 | Guest_IMG | 1,166 | 101 | Guest_IMG | 0,253 | 1,166 | 0,253 |
| 102 | Guest_IMG | 0,177 | 102 | Guest_IMG | 0,24 | 0,177 | 0,24 |
| 103 | Guest_IMG | 0,704 | 103 | Guest_IMG | 0,584 | 0,704 | 0,584 |
| 104 | Guest_IMG | 1,085 | 104 | Guest_IMG | 0,327 | 1,085 | 0,327 |
| 105 | Guest_IMG | 1,05 | 105 | Guest_IMG | 0,562 | 1,05 | 0,562 |
| 106 | Guest_IMG | 0,659 | 106 | Guest_IMG | 1,838 | 0,659 | 1,838 |
| 107 | Guest_IMG | 0,577 | 107 | Guest_IMG | 1,344 | 0,577 | 1,344 |
| 108 | Guest_IMG | 0,744 | 108 | Guest_IMG | 0,544 | 0,744 | 0,544 |
| 109 | Guest_IMG | 0,827 | 109 | Guest_IMG | 1,131 | 0,827 | 1,131 |
| 110 | Guest_IMG | 0,558 | 110 | Guest_IMG | 0,232 | 0,558 | 0,232 |
| 111 | Guest_IMG | 1,512 | 111 | Guest_IMG | 0,856 | 1,512 | 0,856 |
| 112 | Guest_IMG | 0,361 | 112 | Guest_IMG | 0,569 | 0,361 | 0,569 |
| 113 | Guest_IMG | 0,492 | 113 | Guest_IMG | 0,418 | 0,492 | 0,418 |
| 114 | Guest_IMG | 0,64 | 114 | Guest_IMG | 1,779 | 0,64 | 1,779 |
| 115 | Guest_IMG | 0,507 | 115 | Guest_IMG | 1,297 | 0,507 | 1,297 |
| 116 | Guest_IMG | 0,486 | 116 | Guest_IMG | 1,399 | 0,486 | 1,399 |
| 117 | Guest_IMG | 0,48 | 117 | Guest_IMG | 1,86 | 0,48 | 1,86 |
| 118 | Guest_IMG | 0,856 | 118 | Guest_IMG | 0,157 | 0,856 | 0,157 |
| 119 | Guest_IMG | 0,862 | 119 | Guest_IMG | 1,13 | 0,862 | 1,13 |
| 120 | Guest_IMG | 0,236 | 120 | Guest_IMG | 1,241 | 0,236 | 1,241 |
| 121 | Guest_IMG | 0,802 | 121 | Guest_IMG | 0,883 | 0,802 | 0,883 |
| 122 | Guest_IMG | 2,338 | 122 | Guest_IMG | 0,512 | 2,338 | 0,512 |
| 123 | Guest_IMG | 0,666 | 123 | Guest_IMG | 0,408 | 0,666 | 0,408 |
| 124 | Guest_IMG | 1,132 | 124 | Guest_IMG | 0,634 | 1,132 | 0,634 |
| 125 | Guest_IMG | 0,828 | 125 | Guest_IMG | 0,76 | 0,828 | 0,76 |
| 126 | Guest_IMG | 0,34 | 126 | Guest_IMG | 1,032 | 0,34 | 1,032 |
| 127 | Guest_IMG | 0,699 | 127 | Guest_IMG | 0,416 | 0,699 | 0,416 |
| 128 | Guest_IMG | 0,173 | 128 | Guest_IMG | 0,499 | 0,173 | 0,499 |
| 129 | Guest_IMG | 0,431 | 129 | Guest_IMG | 0,429 | 0,431 | 0,429 |
| 130 | Guest_IMG | 0,489 | 130 | Guest_IMG | 1,057 | 0,489 | 1,057 |
| 131 | Guest_IMG | 0,489 | 131 | Guest_IMG | 0,515 | 0,489 | 0,515 |
| 132 | Guest_IMG | 0,706 | 132 | Guest_IMG | 0,971 | 0,706 | 0,971 |
| 133 | Guest_IMG | 0,552 | 133 | Guest_IMG | 0,618 | 0,552 | 0,618 |
| 134 | Guest_IMG | 0,319 | 134 | Guest_IMG | 0,485 | 0,319 | 0,485 |
| 135 | Guest_IMG | 0,353 | 135 | Guest_IMG | 0,49 | 0,353 | 0,49 |
| 136 | Guest_IMG | 0,729 | 136 | Guest_IMG | 0,625 | 0,729 | 0,625 |
| 137 | Guest_IMG | 0,209 | 137 | Guest_IMG | 0,378 | 0,209 | 0,378 |
| 138 | Guest_IMG | 0,642 | 138 | Guest_IMG | 0,951 | 0,642 | 0,951 |
| 139 | Guest_IMG | 0,45 | 139 | Guest_IMG | 0,48 | 0,45 | 0,48 |
| 140 | Guest_IMG | 0,404 | 140 | Guest_IMG | 1,108 | 0,404 | 1,108 |
| 141 | Guest_IMG | 0,394 | 141 | Guest_IMG | 1,079 | 0,394 | 1,079 |
| 142 | Guest_IMG | 0,441 | 142 | Guest_IMG | 1,796 | 0,441 | 1,796 |
| 143 | Guest_IMG | 0,305 | 143 | Guest_IMG | 0,993 | 0,305 | 0,993 |
| 144 | Guest_IMG | 0,276 | 144 | Guest_IMG | 2,546 | 0,276 | 2,546 |
| 145 | Guest_IMG | 0,447 | 145 | Guest_IMG | 0,48 | 0,447 | 0,48 |

| | | |
|---|---|---|
| 146 | Guest_IMG | 1,194 |
| 147 | Guest_IMG | 0,429 |
| 148 | Guest_IMG | 0,208 |
| 149 | Guest_IMG | 0,249 |
| 150 | Guest_IMG | 0,352 |
| 151 | Guest_IMG | 0,218 |
| 152 | Guest_IMG | 0,41 |
| 1 | Guest_IMG | 0,698 |
| 2 | Guest_IMG | 1,021 |
| 3 | Guest_IMG | 0,498 |
| 4 | Guest_IMG | 0,591 |
| 5 | Guest_IMG | 0,554 |
| 6 | Guest_IMG | 0,41 |
| 7 | Guest_IMG | 0,652 |
| 8 | Guest_IMG | 0,292 |
| 9 | Guest_IMG | 0,613 |
| 10 | Guest_IMG | 0,696 |
| 11 | Guest_IMG | 0,56 |
| 12 | Guest_IMG | 0,352 |
| 13 | Guest_IMG | 0,404 |
| 14 | Guest_IMG | 0,235 |
| 15 | Guest_IMG | 0,935 |
| 16 | Guest_IMG | 0,267 |
| 17 | Guest_IMG | 1,188 |
| 18 | Guest_IMG | 0,138 |
| 19 | Guest_IMG | 0,543 |
| 20 | Guest_IMG | 0,341 |
| 21 | Guest_IMG | 0,319 |
| 22 | Guest_IMG | 0,783 |
| 23 | Guest_IMG | 0,497 |
| 24 | Guest_IMG | 0,482 |
| 25 | Guest_IMG | 0,583 |
| 26 | Guest_IMG | 0,555 |
| 27 | Guest_IMG | 0,281 |
| 28 | Guest_IMG | 0,332 |
| 29 | Guest_IMG | 0,31 |
| 30 | Guest_IMG | 0,374 |
| 31 | Guest_IMG | 0,853 |
| 32 | Guest_IMG | 0,512 |
| 33 | Guest_IMG | 0,371 |
| 34 | Guest_IMG | 0,426 |
| 35 | Guest_IMG | 0,628 |
| 36 | Guest_IMG | 0,548 |
| 37 | Guest_IMG | 0,563 |
| 38 | Guest_IMG | 0,386 |
| 39 | Guest_IMG | 0,411 |
| 40 | Guest_IMG | 1,233 |
| 41 | Guest_IMG | 0,573 |
| 42 | Guest_IMG | 0,555 |
| 43 | Guest_IMG | 1,27 |

| | | |
|---|---|---|
| 146 | Guest_IMG | 1,667 |
| 147 | Guest_IMG | 0,627 |
| 148 | Guest_IMG | 2,088 |
| 149 | Guest_IMG | 0,507 |
| 150 | Guest_IMG | 0,361 |
| 151 | Guest_IMG | 0,679 |
| 152 | Guest_IMG | 0,773 |
| 153 | Guest_IMG | 1,363 |
| 154 | Guest_IMG | 0,537 |
| 155 | Guest_IMG | 0,33 |
| 156 | Guest_IMG | 0,4 |
| 157 | Guest_IMG | 0,673 |
| 158 | Guest_IMG | 0,462 |
| 159 | Guest_IMG | 0,418 |
| 160 | Guest_IMG | 0,982 |
| 161 | Guest_IMG | 1,633 |
| 162 | Guest_IMG | 0,4 |
| 163 | Guest_IMG | 0,364 |
| 164 | Guest_IMG | 1,2 |
| 165 | Guest_IMG | 0,462 |
| 166 | Guest_IMG | 0,498 |
| 167 | Guest_IMG | 0,953 |
| 168 | Guest_IMG | 0,923 |
| 169 | Guest_IMG | 0,496 |
| 170 | Guest_IMG | 0,411 |
| 171 | Guest_IMG | 0,273 |
| 172 | Guest_IMG | 0,559 |
| 173 | Guest_IMG | 0,346 |
| 174 | Guest_IMG | 0,53 |
| 175 | Guest_IMG | 0,235 |
| 176 | Guest_IMG | 0,266 |
| 177 | Guest_IMG | 0,97 |
| 178 | Guest_IMG | 0,384 |
| 179 | Guest_IMG | 0,628 |
| 180 | Guest_IMG | 0,269 |
| 181 | Guest_IMG | 0,117 |
| 182 | Guest_IMG | 0,512 |
| 183 | Guest_IMG | 0,768 |
| 184 | Guest_IMG | 0,837 |
| 185 | Guest_IMG | 0,804 |
| 186 | Guest_IMG | 0,381 |
| 187 | Guest_IMG | 0,677 |
| 188 | Guest_IMG | 0,596 |
| 189 | Guest_IMG | 0,525 |
| 190 | Guest_IMG | 0,556 |
| 191 | Guest_IMG | 0,181 |
| 192 | Guest_IMG | 1,104 |
| 193 | Guest_IMG | 0,533 |
| 194 | Guest_IMG | 0,547 |
| 195 | Guest_IMG | 0,41 |

| | |
|---|---|
| 1,194 | 1,667 |
| 0,429 | 0,627 |
| 0,208 | 2,088 |
| 0,249 | 0,507 |
| 0,352 | 0,361 |
| 0,218 | 0,679 |
| 0,41 | 0,773 |
| 0,698 | 1,363 |
| 1,021 | 0,537 |
| 0,498 | 0,33 |
| 0,591 | 0,4 |
| 0,554 | 0,673 |
| 0,41 | 0,462 |
| 0,652 | 0,418 |
| 0,292 | 0,982 |
| 0,613 | 1,633 |
| 0,696 | 0,4 |
| 0,56 | 0,364 |
| 0,352 | 1,2 |
| 0,404 | 0,462 |
| 0,235 | 0,498 |
| 0,935 | 0,953 |
| 0,267 | 0,923 |
| 1,188 | 0,496 |
| 0,138 | 0,411 |
| 0,543 | 0,273 |
| 0,341 | 0,559 |
| 0,319 | 0,346 |
| 0,783 | 0,53 |
| 0,497 | 0,235 |
| 0,482 | 0,266 |
| 0,583 | 0,97 |
| 0,555 | 0,384 |
| 0,281 | 0,628 |
| 0,332 | 0,269 |
| 0,31 | 0,117 |
| 0,374 | 0,512 |
| 0,853 | 0,768 |
| 0,512 | 0,837 |
| 0,371 | 0,804 |
| 0,426 | 0,381 |
| 0,628 | 0,677 |
| 0,548 | 0,596 |
| 0,563 | 0,525 |
| 0,386 | 0,556 |
| 0,411 | 0,181 |
| 1,233 | 1,104 |
| 0,573 | 0,533 |
| 0,555 | 0,547 |
| 1,27 | 0,41 |

| | | | | | | | |
|---|---|---|---|---|---|---|---|
| 44 | Guest_IMG | 0,508 | 196 | Guest_IMG | 1,356 | 0,508 | 1,356 |
| 45 | Guest_IMG | 0,388 | 197 | Guest_IMG | 0,284 | 0,388 | 0,284 |
| 46 | Guest_IMG | 0,659 | 198 | Guest_IMG | 0,599 | 0,659 | 0,599 |
| 47 | Guest_IMG | 0,781 | 199 | Guest_IMG | 0,628 | 0,781 | 0,628 |
| 48 | Guest_IMG | 0,801 | 200 | Guest_IMG | 0,518 | 0,801 | 0,518 |
| 49 | Guest_IMG | 0,682 | 201 | Guest_IMG | 1,379 | 0,682 | 1,379 |
| 50 | Guest_IMG | 0,448 | 202 | Guest_IMG | 0,786 | 0,448 | 0,786 |
| 51 | Guest_IMG | 0,53 | 203 | Guest_IMG | 0,835 | 0,53 | 0,835 |
| 52 | Guest_IMG | 0,75 | 204 | Guest_IMG | 0,981 | 0,75 | 0,981 |
| 53 | Guest_IMG | 0,49 | 205 | Guest_IMG | 0,682 | 0,49 | 0,682 |
| 54 | Guest_IMG | 0,823 | 206 | Guest_IMG | 0,496 | 0,823 | 0,496 |
| 55 | Guest_IMG | 0,829 | 207 | Guest_IMG | 0,342 | 0,829 | 0,342 |
| 56 | Guest_IMG | 0,494 | 208 | Guest_IMG | 1,949 | 0,494 | 1,949 |
| 57 | Guest_IMG | 0,559 | 209 | Guest_IMG | 0,279 | 0,559 | 0,279 |
| 58 | Guest_IMG | 1,249 | 210 | Guest_IMG | 0,111 | 1,249 | 0,111 |
| 59 | Guest_IMG | 0,462 | 211 | Guest_IMG | 0,57 | 0,462 | 0,57 |
| 60 | Guest_IMG | 0,606 | 212 | Guest_IMG | 0,378 | 0,606 | 0,378 |
| 61 | Guest_IMG | 0,611 | 213 | Guest_IMG | 0,432 | 0,611 | 0,432 |
| 62 | Guest_IMG | 0,995 | 214 | Guest_IMG | 1,322 | 0,995 | 1,322 |
| 63 | Guest_IMG | 0,667 | 215 | Guest_IMG | 0,226 | 0,667 | 0,226 |
| 64 | Guest_IMG | 0,424 | 216 | Guest_IMG | 0,325 | 0,424 | 0,325 |
| 65 | Guest_IMG | 0,967 | 217 | Guest_IMG | 0,676 | 0,967 | 0,676 |
| 66 | Guest_IMG | 0,934 | 218 | Guest_IMG | 0,797 | 0,934 | 0,797 |
| 67 | Guest_IMG | 0,726 | 219 | Guest_IMG | 0,524 | 0,726 | 0,524 |
| 68 | Guest_IMG | 1,32 | 220 | Guest_IMG | 0,762 | 1,32 | 0,762 |
| 69 | Guest_IMG | 0,605 | 221 | Guest_IMG | 0,729 | 0,605 | 0,729 |
| 70 | Guest_IMG | 0,543 | 222 | Guest_IMG | 1,188 | 0,543 | 1,188 |
| 71 | Guest_IMG | 0,442 | 223 | Guest_IMG | 0,552 | 0,442 | 0,552 |
| 72 | Guest_IMG | 0,825 | 224 | Guest_IMG | 0,757 | 0,825 | 0,757 |
| 73 | Guest_IMG | 0,472 | 225 | Guest_IMG | 0,605 | 0,472 | 0,605 |
| 74 | Guest_IMG | 0,483 | 226 | Guest_IMG | 0,61 | 0,483 | 0,61 |
| 75 | Guest_IMG | 0,85 | 227 | Guest_IMG | 1,98 | 0,85 | 1,98 |
| 76 | Guest_IMG | 0,871 | 228 | Guest_IMG | 3,599 | 0,871 | 3,599 |
| 77 | Guest_IMG | 0,417 | 229 | Guest_IMG | 0,383 | 0,417 | 0,383 |
| 78 | Guest_IMG | 0,722 | 230 | Guest_IMG | 0,91 | 0,722 | 0,91 |
| 79 | Guest_IMG | 0,649 | 231 | Guest_IMG | 0,603 | 0,649 | 0,603 |
| 80 | Guest_IMG | 1,013 | 232 | Guest_IMG | 0,718 | 1,013 | 0,718 |
| 81 | Guest_IMG | 0,929 | 233 | Guest_IMG | 1,383 | 0,929 | 1,383 |
| 82 | Guest_IMG | 0,892 | 234 | Guest_IMG | 0,312 | 0,892 | 0,312 |
| 83 | Guest_IMG | 1,14 | 235 | Guest_IMG | 2,233 | 1,14 | 2,233 |
| 84 | Guest_IMG | 1,016 | 236 | Guest_IMG | 0,487 | 1,016 | 0,487 |
| 85 | Guest_IMG | 0,534 | 237 | Guest_IMG | 1,072 | 0,534 | 1,072 |
| 86 | Guest_IMG | 0,562 | 238 | Guest_IMG | 0,665 | 0,562 | 0,665 |
| 87 | Guest_IMG | 0,727 | 239 | Guest_IMG | 0,347 | 0,727 | 0,347 |
| 88 | Guest_IMG | 0,626 | 240 | Guest_IMG | 0,523 | 0,626 | 0,523 |
| 89 | Guest_IMG | 1,519 | 241 | Guest_IMG | 0,495 | 1,519 | 0,495 |
| 90 | Guest_IMG | 0,35 | 242 | Guest_IMG | 0,903 | 0,35 | 0,903 |
| 91 | Guest_IMG | 0,469 | 243 | Guest_IMG | 0,848 | 0,469 | 0,848 |
| 92 | Guest_IMG | 0,652 | 244 | Guest_IMG | 0,252 | 0,652 | 0,252 |
| 93 | Guest_IMG | 0,444 | 245 | Guest_IMG | 1,132 | 0,444 | 1,132 |

| | | | | | | | | |
|---|---|---|---|---|---|---|---|---|
| 94 | Guest_IMG | 0,452 | 246 | Guest_IMG | 0,782 | | 0,452 | 0,782 |
| 95 | Guest_IMG | 1,211 | 247 | Guest_IMG | 0,474 | | 1,211 | 0,474 |
| 96 | Guest_IMG | 0,921 | 248 | Guest_IMG | 0,159 | | 0,921 | 0,159 |
| 97 | Guest_IMG | 0,758 | 249 | Guest_IMG | 0,399 | | 0,758 | 0,399 |
| 98 | Guest_IMG | 0,292 | 250 | Guest_IMG | 0,478 | | 0,292 | 0,478 |

| | | |
|---|---|---|
| mean | 0,60554 | 0,684348 |
| SD | 0,29983 | 0,460885 |
| n | 250 | 250 |
| SEM | 0,018963 | 0,029149 |
| | | |
| t probe | p= | 0,023863 |
