# Supplementary material for: Cardiac effects of OPA1 protein promotion in a transgenic animal model
Source: PLoS One. 2024 Nov 21;19(11):e0310394. doi: 10.1371/journal.pone.0310394 (PMC11581344; doi:10.1371/journal.pone.0310394)

Supplementary information for Figure 6.

| WT | | Average branch length | TG | | Average branch length |
|---|---|---|---|---|---|
| 1st cell | 1 | 4,552 | 1st cell | 1 | 1,414 |
| | 2 | 2 | | 2 | 5 |
| | 3 | 2 | | 3 | 12 |
| | 4 | 13,071 | | 4 | 13,071 |
| | 5 | 13,243 | | 5 | 19,628 |
| | 6 | 4,414 | | 6 | 7,657 |
| | 7 | 9,243 | | 7 | 0 |
| | 8 | 4,886 | | 8 | 3,414 |
| | 9 | 2 | | 9 | 3,828 |
| | 10 | 21,071 | | 10 | 1 |
| | 11 | 0 | | 11 | 4,552 |
| | 12 | 0 | | 12 | 3 |
| | 13 | 15,243 | | 13 | 9,243 |
| | 14 | 7,657 | | 14 | 0 |
| | 15 | 17,657 | | 15 | 6,657 |
| | 16 | 1 | | 16 | 5,414 |
| | 17 | 39,799 | | 17 | 2 |
| | 18 | 21,899 | | 18 | 7,74 |
| | 19 | 72,113 | | 19 | 6,243 |
| | 20 | 27,314 | | 20 | 0 |
| | 21 | 12,657 | | 21 | 0 |
| | 22 | 14,811 | | 22 | 16,728 |
| | 23 | 11,657 | | 23 | 9,754 |
| 2nd cell | 24 | 0 | 2nd cell | 24 | 4,828 |
| | 25 | 1 | | 25 | 5,414 |
| | 26 | 0 | | 26 | 0 |
| | 27 | 1 | | 27 | 6 |
| | 28 | 12,485 | | 28 | 5,932 |
| | 29 | 18,704 | | 29 | 15,152 |
| | 30 | 66,284 | | 30 | 51,698 |
| | 31 | 8,414 | | 31 | 17,728 |
| | 32 | 7 | | 32 | 0 |
| | 33 | 7 | | 33 | 1 |
| | 34 | 0 | | 34 | 1 |
| | 35 | 2,414 | | 35 | 11 |
| | 36 | 1,414 | | 36 | 4,414 |
| | 37 | 2 | | 37 | 13,071 |
| | 38 | 4,219 | | 38 | 0 |
| | 39 | 12,243 | | 39 | 4,243 |
| | 40 | 6,768 | | 40 | 24,556 |
| | 41 | 2 | | 41 | 15,374 |
| | 42 | 12,324 | | 42 | 5,414 |
| | 43 | 0 | | 43 | 0 |
| | 44 | 7,024 | | 44 | 0 |
| | 45 | 13,761 | | 45 | 10,243 |
| | 46 | 11,657 | | 46 | 9,162 |
| | 47 | 2 | | 47 | 9,047 |

| | | | | | |
|---|---|---|---|---|---|
| | 48 | 3,297 | | 48 | 24,556 |
| | 49 | 1 | | 49 | 2 |
| 3rd cell | 50 | 1 | 3rd cell | 50 | 12,243 |
| | 51 | 5,828 | | 51 | 1 |
| | 52 | 5,414 | | 52 | 12,657 |
| | 53 | 10,105 | | 53 | 0 |
| | 54 | 2,276 | | 54 | 2 |
| | 55 | 8 | | 55 | 8,414 |
| | 56 | 16,071 | | 56 | 8,633 |
| | 57 | 0 | | 57 | 7,414 |
| | 58 | 12,071 | | 58 | 3 |
| | 59 | 0 | | 59 | 23,349 |
| | 60 | 1 | | 60 | 18,485 |
| | 61 | 6,414 | | 61 | 0 |
| | 62 | 21,142 | | 62 | 14,485 |
| | 63 | 11,243 | | 63 | 3 |
| | 64 | 21,142 | | 64 | 2,414 |
| | 65 | 3 | | 65 | 0 |
| | 66 | 11,243 | | 66 | 22,899 |
| | 67 | 11,657 | | 67 | 3,414 |
| | 68 | 0 | | 68 | 15,485 |
| | 69 | 12,071 | | 69 | 2 |
| 4th cell | 70 | 12,899 | 4th cell | 70 | 1 |
| | 71 | 26,971 | | 71 | 17,414 |
| | 72 | 1 | | 72 | 6,828 |
| | 73 | 3,414 | | 73 | 2 |
| | 74 | 3,414 | | 74 | 1 |
| | 75 | 3 | | 75 | 9,243 |
| | 76 | 44,799 | | 76 | 10,243 |
| | 77 | 3,414 | | 77 | 0 |
| | 78 | 5,438 | | 78 | 1 |
| | 79 | 1 | | 79 | 1 |
| | 80 | 13,657 | | 80 | 2 |
| | 81 | 0 | | 81 | 1 |
| | 82 | 4,828 | | 82 | 13,071 |
| | 83 | 1 | | 83 | 1 |
| | 84 | 1 | | 84 | 14,657 |
| | 85 | 43,627 | | 85 | 16,071 |
| | 86 | 9,071 | | 86 | 11,657 |
| | 87 | 4,828 | | 87 | 21,657 |
| | 88 | 1,414 | | 88 | 0 |
| | 89 | 0 | | 89 | 12,519 |
| 5th cell | 90 | 15,071 | 5th cell | 90 | 1 |
| | 91 | 1 | | 91 | 22,314 |
| | 92 | 11,071 | | 92 | 1 |
| | 93 | 13,314 | | 93 | 6,414 |
| | 94 | 5,69 | | 94 | 4,414 |
| | 95 | 2,414 | | 95 | 1 |
| | 96 | 0 | | 96 | 15,828 |
| | 97 | 4 | | 97 | 0 |

| | | | | | |
|---|---|---|---|---|---|
| | 98 | 2 | | 98 | 2 |
| | 99 | 7,828 | | 99 | 17,728 |
| | 100 | 0 | | 100 | 4,828 |
| | 101 | 2,414 | | 101 | 4,69 |
| | 102 | 1 | | 102 | 1,414 |
| | 103 | 0 | | 103 | 16,485 |
| | 104 | 11,266 | | 104 | 3,414 |
| | 105 | 1 | | 105 | 0 |
| | 106 | 0 | | 106 | 0 |
| | 107 | 1 | | 107 | 66,77 |
| | 108 | 14,29 | | 108 | 18,556 |
| | 109 | 0 | | 109 | 2 |
| 6th cell | 110 | 18,899 | 6th cell | 110 | 8,69 |
| | 111 | 6,828 | | 111 | 5 |
| | 112 | 7,414 | | 112 | 1 |
| | 113 | 1 | | 113 | 2,414 |
| | 114 | 9,519 | | 114 | 0 |
| | 115 | 2 | | 115 | 0 |
| | 116 | 17,142 | | 116 | 4 |
| | 117 | 0 | | 117 | 3,121 |
| | 118 | 7,828 | | 118 | 1 |
| | 119 | 13,798 | | 119 | 1 |
| | 120 | 6,828 | | 120 | 4,219 |
| | 121 | 12,657 | | 121 | 1 |
| | 122 | 2,414 | | 122 | 4,828 |
| | 123 | 2 | | 123 | 2,414 |
| | 124 | 26,142 | | 124 | 6,414 |
| | 125 | 7,243 | | 125 | 8,243 |
| | 126 | 6,243 | | 126 | 0 |
| | 127 | 10,933 | | 127 | 2,805 |
| | 128 | 0 | | 128 | 0 |
| | 129 | 20,071 | | 129 | 14,081 |
| 7th cell | 130 | 2 | 7th cell | 130 | 3,414 |
| | 131 | 0 | | 131 | 0 |
| | 132 | 5,759 | | 132 | 0 |
| | 133 | 3,609 | | 133 | 0 |
| | 134 | 3,414 | | 134 | 17,657 |
| | 135 | 1 | | 135 | 5,456 |
| | 136 | 0 | | 136 | 20,657 |
| | 137 | 24,728 | | 137 | 0 |
| | 138 | 3,414 | | 138 | 1 |
| | 139 | 1 | | 139 | 17,142 |
| | 140 | 5,414 | | 140 | 0 |
| | 141 | 0 | | 141 | 2 |
| | 142 | 15,657 | | 142 | 8,657 |
| | 143 | 0 | | 143 | 3 |
| | 144 | 10,828 | | 144 | 3,414 |
| | 145 | 0 | | 145 | 6,243 |
| | 146 | 3 | | 146 | 3 |
| | 147 | 3 | | 147 | 0 |

| | | |
|---|---|---|
| | 148 | 0 |
| | 149 | 15,828 |
| 8th cell | 150 | 1 |
| | 151 | 0 |
| | 152 | 7,828 |
| | 153 | 9,657 |
| | 154 | 0 |
| | 155 | 1,414 |
| | 156 | 11,828 |
| | 157 | 18,071 |
| | 158 | 21,899 |
| | 159 | 0 |
| | 160 | 0 |
| | 161 | 0 |
| | 162 | 9,657 |
| | 163 | 1 |
| | 164 | 2 |
| | 165 | 9,803 |
| | 166 | 2 |
| | 167 | 0 |
| | 168 | 3,414 |
| | 169 | 13,761 |
| 9th cell | 170 | 15,071 |
| | 171 | 8,462 |
| | 172 | 3 |
| | 173 | 0 |
| | 174 | 0 |
| | 175 | 0 |
| | 176 | 0 |
| | 177 | 0 |
| | 178 | 1 |
| | 179 | 0 |
| | 180 | 22,542 |
| | 181 | 2,414 |
| | 182 | 10,105 |
| | 183 | 2,609 |
| | 184 | 0 |
| | 185 | 12,071 |
| | 186 | 0 |
| | 187 | 6,414 |
| | 188 | 8,828 |
| | 189 | 0 |
| 10th cell | 190 | 4,414 |
| | 191 | 19,728 |
| | 192 | 3 |
| | 193 | 21,142 |
| | 194 | 8,828 |
| | 195 | 11,657 |
| | 196 | 0 |
| | 197 | 6 |

| | | |
|---|---|---|
| | 148 | 7,657 |
| | 149 | 4 |
| 8th cell | 150 | 9 |
| | 151 | 0 |
| | 152 | 12,828 |
| | 153 | 5,828 |
| | 154 | 0 |
| | 155 | 1 |
| | 156 | 20,314 |
| | 157 | 16,071 |
| | 158 | 2 |
| | 159 | 0 |
| | 160 | 1 |
| | 161 | 0 |
| | 162 | 7,414 |
| | 163 | 14,071 |
| | 164 | 44,627 |
| | 165 | 28,899 |
| | 166 | 0 |
| | 167 | 8,071 |
| | 168 | 32,142 |
| | 169 | 18,728 |
| 9th cell | 170 | 4,414 |
| | 171 | 0 |
| | 172 | 5,731 |
| | 173 | 16,957 |
| | 174 | 0 |
| | 175 | 36,385 |
| | 176 | 0 |
| | 177 | 15,623 |
| | 178 | 5,414 |
| | 179 | 12,828 |
| | 180 | 17,128 |
| | 181 | 0 |
| | 182 | 1 |
| | 183 | 15,899 |
| | 184 | 15,399 |
| | 185 | 45,556 |
| | 186 | 4,414 |
| | 187 | 1 |
| 10th cell | 188 | 9,071 |
| | 189 | 5 |
| | 190 | 6 |
| | 191 | 22,657 |
| | 192 | 3 |
| | 193 | 7,657 |
| | 194 | 3,828 |
| | 195 | 2 |
| | 196 | 1 |

| | | |
|---|---|---|
| | 198 | 12,071 |
| | 199 | 11,243 |
| | 200 | 7,828 |

| | WT | TG |
|---|---|---|
| Mean | 7,747365 | 7,794704 |
| SD | 10,10793 | 9,776597 |
| N | 200 | 196 |
| SEM | 0,714738 | 0,698328 |
| | | |
| T-probe | 0,962252 | |

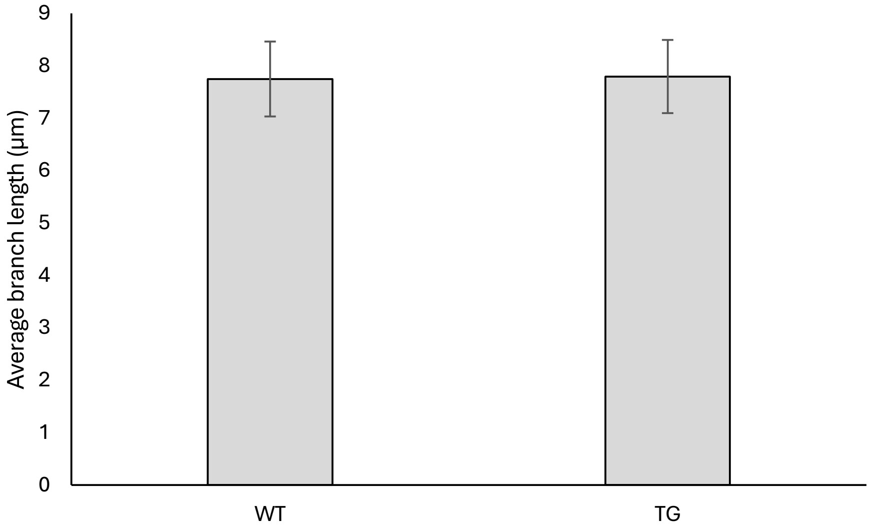

Supplement: S5 Fig — (PDF) [file pone.0310394.s005.pdf]
