# Supplementary material for: Cardiac effects of OPA1 protein promotion in a transgenic animal model
Source: PLoS One. 2024 Nov 21;19(11):e0310394. doi: 10.1371/journal.pone.0310394 (PMC11581344; doi:10.1371/journal.pone.0310394)

Supplementary information for Figure 8.

	WT		TG		WT+FCCP	
	Red	Green	Red	Green	Red	Green
1	78,17	14,69	86,10	14,00	12,05	84,81
2	69,86	17,14	51,88	48,45	23,87	72,62
3	81,89	14,52	43,36	53,57	15,91	80,43
4	77,45	21,93	44,50	50,82	18,26	78,24
5	50,43	45,72	68,83	24,77	32,11	66,04
6	78,15	23,31	42,36	54,70	19,15	80,85
7	55,11	42,51	80,16	18,49	30,24	68,66
8	76,14	23,83	37,97	62,28	5,69	94,07
9	74,17	25,15	75,78	24,08	11,14	87,27
10	72,21	26,48	53,59	46,87	8,59	91,08
11	70,24	27,80	41,39	58,66	22,04	76,89
12	68,27	29,12	69,20	27,45	12,50	84,51
13	66,31	30,44	47,01	46,25	10,95	86,14
14	64,34	31,76	74,81	25,04	35,40	59,76
15	55,37	43,09	62,62	33,83	21,85	77,39
16	62,41	34,41	60,43	39,62	24,30	75,01
17	61,44	35,73	38,24	61,41	24,75	72,64
18	63,47	36,05	36,04	60,21	18,20	81,26
19	60,51	38,37	33,85	55,00	35,65	64,09
20	59,54	39,70	51,66	47,21	16,10	81,52
21	60,57	39,02	49,47	43,42	12,55	83,14
22	68,61	28,61	47,27	44,62	17,00	81,77
23	66,64	26,64	45,08	45,83	13,45	86,39
24	54,67	44,98	72,89	27,04	31,91	66,02
**Mean**	**66,49734286**	**30,87496984**	**54,77067**	**42,23475**	**19,74**	**78,35821**
**SD**	**8,52878193**	**9,23340902**	**15,41859**	**14,48046**	**8,592769**	**8,805528**
**Count**	**24**	**24**	**24**	**24**	**24**	**24**
SEM	*1,74093032*	*1,88476172*	*3,14731*	*2,95581*	*1,75399*	*1,79742*

	red	green
**WT vs TG**	0,00209797	0,002220569
**WT vs WT+FCCP**	2,44985E-23	1,11113E-22
**TG vs WT+FCCP**	9,84989E-13	1,00999E-13

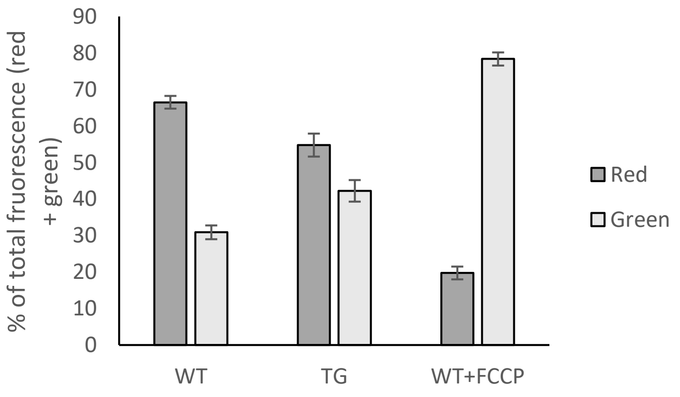

Supplement: S6 Fig — (PDF) [file pone.0310394.s006.pdf]
