# Supplementary material for: Cardiac effects of OPA1 protein promotion in a transgenic animal model
Source: PLoS One. 2024 Nov 21;19(11):e0310394. doi: 10.1371/journal.pone.0310394 (PMC11581344; doi:10.1371/journal.pone.0310394)
Supplement: S7 Fig — (PDF) [file pone.0310394.s007.pdf]

Supplementary information for Figure 8.

| | Time | Group Name: WT | | | Group Name: WT | | |
|---|---|---|---|---|---|---|---|
| | | B01 | C01 | D01 | B01 | C01 | D01 |
| WT | 1,59 | 85,4958 | 114,0189 | 87,0894 | 78,8942 | 82,6619 | 94,8599 |
| | 8,19 | 80,9886 | 106,8801 | 82,9106 | 75,6927 | 78,8876 | 90,5404 |
| | 14,78 | 79,6291 | 103,7307 | 80,233 | 73,1465 | 76,7085 | 88,2522 |
| | 21,46 | 38,1528 | 42,9359 | 35,4128 | 33,7438 | 32,8522 | 35,4026 |
| | 28,06 | 37,4694 | 43,4562 | 35,7248 | 33,0317 | 33,1786 | 35,1395 |
| | 34,65 | 37,9994 | 43,3058 | 35,1742 | 33,9658 | 33,2363 | 36,1184 |
| | 41,32 | 213,6152 | 295,5257 | 208,4576 | 182,7389 | 220,6495 | 236,913 |
| | 47,93 | 198,4841 | 251,9305 | 194,4193 | 180,3701 | 198,5386 | 209,9575 |
| | 54,53 | 194,2755 | 245,0024 | 191,2757 | 166,29 | 193,775 | 205,8814 |
| | 61,20 | 22,4466 | 26,2987 | 22,7653 | 21,8354 | 18,9224 | 19,2316 |
| | 67,81 | 23,9912 | 29,8828 | 23,0089 | 21,8399 | 20,8737 | 22,1036 |
| | 74,41 | 25,0304 | 28,4425 | 22,7225 | 22,0896 | 21,0377 | 22,9772 |

| | Time | Group Name: OPA TG | | | Group Name: OPA TG | | |
|---|---|---|---|---|---|---|---|
| | | E01 | F01 | G01 | E01 | F01 | G01 |
| TG | 1,59 | 83,5763 | 87,3309 | 72,4728 | 55,6581 | 43,6215 | 79,8306 |
| | 8,19 | 78,5401 | 87,8113 | 69,62 | 52,105 | 41,9781 | 75,0556 |
| | 14,78 | 75,9374 | 89,2635 | 67,6422 | 50,4917 | 40,3404 | 72,5349 |
| | 21,46 | 30,1021 | 34,6158 | 29,858 | 24,532 | 24,2308 | 28,2715 |
| | 28,06 | 29,1688 | 33,7349 | 29,0185 | 22,3568 | 22,917 | 27,2151 |
| | 34,65 | 27,0231 | 30,748 | 26,7212 | 20,5036 | 20,4334 | 25,1809 |
| | 41,32 | 165,6439 | 237,8148 | 144,5905 | 138,8311 | 121,1269 | 183,803 |
| | 47,93 | 149,0529 | 203,3846 | 132,3618 | 142,21 | 112,4851 | 155,5165 |
| | 54,53 | 144,7462 | 206,122 | 128,8097 | 130,1896 | 110,2777 | 152,8649 |
| | 61,20 | 15,6216 | 17,1499 | 14,0035 | 12,6853 | 11,527 | 11,2137 |
| | 67,81 | 17,5094 | 19,9684 | 15,6666 | 13,0991 | 12,999 | 14,3587 |
| | 74,41 | 17,9897 | 21,04 | 15,9292 | 12,3882 | 12,5686 | 14,9852 |

| | Mean | | SD | | SEM | |
|---|---|---|---|---|---|---|
| | WT | TG | WT | TG | WT | TG |
| 1,59 | 90,50335 | 70,41503 | 12,6882 | 17,25058 | 5,179935 | 7,042522 |
| 8,19 | 85,98333 | 67,51835 | 11,38488 | 17,23012 | 4,647857 | 7,034168 |
| 14,78 | 83,61667 | 66,03502 | 11,05224 | 17,80033 | 4,512056 | 7,266955 |
| 21,46 | 36,41668 | 28,6017 | 3,67152 | 3,892946 | 1,498892 | 1,589289 |
| 28,06 | 36,33337 | 27,40185 | 3,864571 | 4,276552 | 1,577705 | 1,745895 |
| 34,65 | 36,63332 | 25,1017 | 3,67254 | 4,028327 | 1,499308 | 1,644558 |
| 41,32 | 226,3167 | 165,3017 | 38,24548 | 41,66923 | 15,61365 | 17,01139 |
| 47,93 | 205,6167 | 149,1685 | 24,60714 | 30,5164 | 10,04582 | 12,45827 |
| 54,53 | 199,4167 | 145,5017 | 25,85252 | 33,11622 | 10,55425 | 13,51964 |
| 61,20 | 21,91667 | 13,70017 | 2,697593 | 2,350306 | 1,101288 | 0,959509 |
| 67,81 | 23,61668 | 15,6002 | 3,247898 | 2,731069 | 1,325949 | 1,114954 |
| 74,41 | 23,71665 | 15,81682 | 2,661533 | 3,317035 | 1,086566 | 1,354174 |

**WT**

| | | | | | | |
|---|---|---|---|---|---|---|
| **Basal respiration** | 57,1925 | 77,432 | 57,4105 | 51,4111 | 57,7861 | 69,0206 |
| **ATP production** | 42,0596 | 60,9048 | 44,9588 | 40,1148 | 43,8563 | 52,8597 |
| **Maximal respiration** | 191,1687 | 269,227 | 185,7451 | 161,0036 | 201,6371 | 217,6814 |
| **Spare respiratory capacity** | 133,9862 | 191,795 | 128,3246 | 109,6024 | 143,841 | 148,6608 |

**TG**

| | | | | | | |
|---|---|---|---|---|---|---|
| **Basal respiration** | 60,3258 | 72,1148 | 53,6285 | 38,1034 | 28,8134 | 61,3212 |
| **ATP production** | 48,9143 | 58,5256 | 40,9108 | 29,9881 | 19,907 | 47,3542 |
| **Maximal respiration** | 150,0223 | 220,6661 | 130,5969 | 129,8108 | 109,5798 | 172,5893 |
| **Spare respiratory capacity** | 89,7065 | 148,5513 | 76,9584 | 91,7173 | 80,7764 | 111,268 |

| | **Mean** | | **SD** | | **SEM** | | **p value** |
|---|---|---|---|---|---|---|---|
| | **WT** | **TG** | **WT** | **TG** | **WT** | **TG** | |
| **Basal respiration** | 61,7088 | 52,38452 | 9,601916 | 16,08296 | 3,919966 | 6,56584 | 0,250694 |
| **ATP production** | 47,459 | 40,93333 | 7,90289 | 13,97366 | 3,226341 | 5,704725 | 0,34288 |
| **Maximal respiration** | 204,4105 | 152,2109 | 36,85889 | 39,7304 | 15,04758 | 16,21987 | 0,04 |
| **Spare respiratory capacity** | 142,7017 | 99,82965 | 27,6593 | 26,67974 | 11,29186 | 10,89196 | 0,021098 |
