# Supplementary material for: Cardiac effects of OPA1 protein promotion in a transgenic animal model
Source: PLoS One. 2024 Nov 21;19(11):e0310394. doi: 10.1371/journal.pone.0310394 (PMC11581344; doi:10.1371/journal.pone.0310394)
Supplement: S8 Fig — (PDF) [file pone.0310394.s008.pdf]

	Mitochondrial DNA	Nuclear DNA			Mitochondrial DNA	Nuclear DNA			Average Number of Copies	Average Number of Copies Fold change
	**trLEV Ct**	**BECN1 Ct**	**ΔCt**	**2 ^ΔCt**	**12s Ct**	**NEB Ct**	**ΔCt**	**2 ^ΔCt**		
WT	18,8	27	8,2	294,066779	17,1	24,1	7	128	211,033	0,47871622
WT	18,9	26,5	7,6	194,011721	17,3	24,9	7,6	194,011721	194,012	0,44010362
WT	19,3	28,8	9,5	724,077344	18,2	25,5	7,3	157,586485	440,832	1
WT	19,7	29	9,3	630,34594	17,9	26	8,1	274,374006	452,36	1,02615069
WT	18,1	28,2	10,1	1097,49603	19,1	29,1	10	1024	1060,75	2,40624142
WT	18,5	28	9,5	724,077344	18,4	27,9	9,5	724,077344	724,077	1,64252478
WT	19	27,1	8,1	274,374006	17,4	26,4	9	512	393,187	0,89192046
WT	19,3	26,7	7,4	168,897013	17,7	26,8	9,1	548,748013	358,823	0,81396673
WT	17,4	25,9	8,5	362,038672	16,8	25,6	8,8	445,721888	403,88	0,9161775
WT	17,8	26,3	8,5	362,038672	17,2	26,1	8,9	477,712892	419,876	0,95246231
WT	17,7	26,1	8,4	337,794025	17	25,3	8,3	315,17297	326,483	0,74060767
WT	18	26,6	8,6	388,023441	17,3	24,9	7,6	194,011721	291,018	0,66015543
WT	18,3	26,8	8,5	362,038672	18,5	27	8,5	362,038672	362,039	0,82126239
WT	18,7	26,2	7,5	181,019336	18	26,8	8,8	445,721888	313,371	0,7108619
WT	18,4	27	8,6	388,023441	17,6	26,6	9	512	450,012	1,02082382
WT	19	27,2	8,2	294,066779	17,8	27,2	9,4	675,58805	484,827	1,09980108
TG	17,7	26	8,3	315,17297	17	25,2	8,2	294,066779	304,62	0,69101139
TG	18	26,4	8,4	337,794025	17,2	25,9	8,7	415,873227	376,834	0,85482383
TG	18,5	25,7	7,2	147,033389	16,9	26,4	9,5	724,077344	435,555	0,98803048
TG	18,8	26,2	7,4	168,897013	16,5	26	9,5	724,077344	446,487	1,01282862
TG	18,1	25,8	7,7	207,936613	17,8	27,3	9,5	724,077344	466,007	1,05710808
TG	18	25,7	7,7	207,936613	17,2	27,5	10,3	1260,69188	734,314	1,66574656
TG	19,1	26,6	7,5	181,019336	17,1	26,7	9,6	776,046882	478,533	1,08552283
TG	18,7	26,9	8,2	294,066779	17,4	26,6	9,2	588,133558	441,1	1,00060852
TG	19,6	28,3	8,7	415,873227	18	28,3	10,3	1260,69188	838,283	1,90159225
TG	19	27,8	8,8	445,721888	18,2	27,9	9,7	831,746454	638,734	1,44892906
TG	18,4	25,8	7,4	168,897013	17,4	27,4	10	1024	596,449	1,35300664
TG	17,8	25,4	7,6	194,011721	17,2	26,9	9,7	831,746454	512,879	1,16343457
TG	18,4	26,5	8,1	274,374006	16,9	25,7	8,8	445,721888	360,048	0,81674655
TG	18,1	27	8,9	477,712892	16,7	25,3	8,6	388,023441	432,868	0,98193473
TG	18,9	27,4	8,5	362,038672	17	27	10	1024	693,019	1,57207161
TG	18,7	26,9	8,2	294,066779	17,3	26,4	9,1	548,748013	421,407	0,95593668

		**MEAN**	**SD**	**N**	**SEM**	**T-probe**
**WT**	Average Number of Copies	430,411089	207,756524	16	51,9391	0,216
	Average Number of Copies Fold change	0,976361	0,47128286	16	0,11782	
**TG**	Average Number of Copies	511,071107	148,113615	16	37,0284	0,216
	Average Number of Copies Fold change	1,15933327	0,3359866	16	0,084	

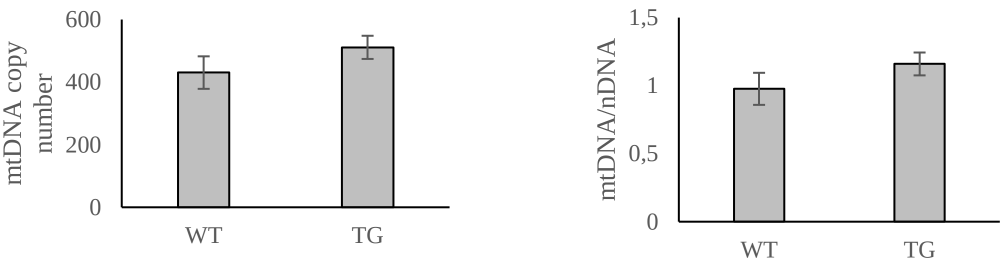
600
400
200
0
mtDNA copy number
WT
TG
1,5
1
0,5
0
mtDNA/nDNA
WT
TG
