# Supplementary material for: Cardiac effects of OPA1 protein promotion in a transgenic animal model
Source: PLoS One. 2024 Nov 21;19(11):e0310394. doi: 10.1371/journal.pone.0310394 (PMC11581344; doi:10.1371/journal.pone.0310394)
Supplement: S9 Fig — (PDF) [file pone.0310394.s009.pdf]

Supplementary information for Figure 11.

VDAC

			Area	*Percent*	*Optical density (%)*		**WT**	**TG**
gel1	**WT**	1	9858,61	14,751	59,004		59,004	63,876
		2	8238,903	12,328	49,312		49,312	68,376
		3	6380,782	9,547	38,188		38,188	42,892
		4	7871,196	11,778	47,112		47,112	31,244
	**TG**	5	10672,2	15,969	63,876		61,724	48,308
		6	11424,02	17,094	68,376		31,224	71,896
		7	7166,66	10,723	42,892		51,728	35,344
		8	5220,004	7,811	31,244		41,608	58,172
gel2	**WT**	1	10094,61	15,431	61,724	mean	47,4875	52,5135
		2	5106,175	7,806	31,224	SD	10,29778	15,33375
		3	8459,539	12,932	51,728	n	8	8
		4	6804,589	10,402	41,608	SEM	3,640815	5,421301
	**TG**	5	7900,539	12,077	48,308	P=	0,454309	
		6	11757,78	17,974	71,896			
		7	5780,004	8,836	35,344			
		8	9513,489	14,543	58,172			

OMA1

			Area	*Percent*	*Optical density (%)*			
gel1	**WT**	1	7388,581	6,311	25,244			
		2	17458,64	14,913	59,652		**WT**	**TG**
		3	28140,29	24,037	96,148		25,244	39,656
		4	13983,26	11,944	47,776		59,652	41,98
	**TG**	5	11606,84	9,914	39,656		96,148	57,52
		6	12286,84	10,495	41,98		47,776	32,024
		7	16834,79	14,38	57,52		44,632	43,884
		8	9372,945	8,006	32,024		58,776	43,824
							41,112	49,86
gel2	**WT**	1	19882,6	11,158	44,632		40,168	77,74
		2	26184,57	14,694	58,776			
		3	18315,25	10,278	41,112	mean	51,6885	48,311
		4	17894,58	10,042	40,168	SD	21,04178	14,00642
	**TG**	5	19549,82	10,971	43,884	n	8	8
		6	19522,52	10,956	43,824	SEM	7,439392	4,952019
		7	22212,12	12,465	49,86	P=	0,711151	
		8	34631,57	19,435	77,74			

YME1L1

			Area	*Percent*	*Optical density (%)*			
gel1	**WT**	1	7708,903	10,986	43,944		**WT**	**TG**
		2	10120,92	14,424	57,696		43,944	43,776
		3	13224,12	18,846	75,384		57,696	47,588
		4	9359,681	13,339	53,356		75,384	45,7
	**TG**	5	7679,56	10,944	43,776		53,356	32,56
		6	8347,681	11,897	47,588		52,788	57,084
		7	8016,56	11,425	45,7		69,256	34,212
		8	5711,61	8,14	32,56		60,776	31,796
							61,064	33,028
gel2	**WT**	1	12141,82	13,197	52,788			
		2	15929,72	17,314	69,256	mean	59,283	40,718
		3	13978,6	15,194	60,776	SD	9,850466	9,23374
		4	14045,31	15,266	61,064	n	8	8
	**TG**	5	13129,65	14,271	57,084	SEM	3,482666	3,26462
		6	7868,924	8,553	34,212	P=	0,001636	
		7	7313,146	7,949	31,796			
		8	7596,56	8,257	33,028			

FLAG

			Area	*Percent*	*Optical density (%)*			
gel1	**WT**	1	5713,619	3,909	15,636		**WT**	**TG**
		2	5452,548	3,73	14,92		15,636	90,276
		3	3404,062	2,329	9,316		14,92	91,376
		4	4026,891	2,755	11,02		9,316	98,632
	**TG**	5	32988,77	22,569	90,276		11,02	68,828
		6	33391,35	22,844	91,376		2,044	119
		7	36042,82	24,658	98,632		3,38	99,852
		8	25151,14	17,207	68,828		6,392	70,688
							10,484	88,16
gel2	**WT**	1	753,849	0,511	2,044			
		2	1247,092	0,845	3,38	mean	9,149	90,8515
		3	2359,406	1,598	6,392	SD	4,957819	16,18348
		4	3868,891	2,621	10,484	n	8	8
	**TG**	5	43913,52	29,75	119	SEM	1,752854	5,721724
		6	36847,13	24,963	99,852	P=	1,76E-09	
		7	26084,63	17,672	70,688			
		8	32532,96	22,04	88,16			

BNIP3

			Area	*Percent*	*Optical density (%)*			
gel1	**WT**	1	22828,37	16,993	33,986		**WT**	**TG**
		2	9452,358	7,036	14,072		33,986	26,462
		3	14816,22	11,029	22,057		14,072	36,627
		4	17801,34	13,251	26,502		22,057	27,328
	**TG**	5	17774,66	13,231	26,462		26,502	12,967
		6	24602,67	18,313	36,627		32,186	37,239
		7	18356,27	13,664	27,328		23,518	36,378
		8	8709,952	6,483	12,967		21,017	0,024
							27,789	21,848
gel2	**WT**	1	18150,84	16,093	32,186			
		2	13262,74	11,759	23,518	mean	25,14088	24,85913
		3	11852,39	10,509	21,017	SD	6,418785	13,09507
		4	15671,4	13,895	27,789	n	8	8
	**TG**	5	21000,68	18,62	37,239	SEM	2,269383	4,629807
		6	20514,9	18,189	36,378	P=	0,957194	
		7	13,4885	0,012	0,024			
		8	12320,83	10,924	21,848			

OPA1

			Area	*Optical density (%)*			
gel1	**WT**	1	4751,046	93,90695		**WT**	**TG**
		2	4159,782	82,2203		93,90695	772,6525
		3	2825,053	55,83867		82,2203	729,4787
		4	5651,924	111,7133		55,83867	570,5206
	**TG**	5	39090,91	772,6525		111,7133	450,6808
		6	36906,61	729,4787		91,88741	770,5784
		7	28864,42	570,5206		80,97165	728,8781
		8	22801,35	450,6808		56,62603	571,7219
						107,833	438,7936
gel2	**WT**	1	4648,871	91,88741			
		2	4096,609	80,97165	mean	85,12466	629,1631
		3	2864,888	56,62603	SD	20,84298	139,0933
		4	5455,607	107,833	n	8	8
	**TG**	5	38985,97	770,5784	SEM	7,369107	49,17692
		6	36876,22	728,8781	P=	3,03E-08	
		7	28925,2	571,7219	P=	<0,001	
		8	22199,94	438,7936			

L-OPA/S-OPA

			Long-OPA Area	Short-OPA Area	*Optical density (%)* L-OPA/S-OPA	
gel1						
	**WT**	1	2796,205	1954,841	1,430401	
		2	2611,012	1548,77	1,685862	**WT**
		3	1306,991	1518,062	0,86096	1,430401

		4	2793,134	2858,79	0,977034			1,685862
	**TG**	5	24808,29	14282,62	1,736957			0,86096
		6	27744,99	9161,619	3,028395			0,977034
		7	20706,34	8158,083	2,538138			1,320466
		8	13898,25	8903,104	1,561057			1,561412
								0,928656
gel2			Long-OPA	Short-OPA	L-OPA/S-OPA			0,954475
	**WT**	1	2645,45	2003,421	1,320466			
		2	2497,253	1599,356	1,561412		mean	1,214908
		3	1379,456	1485,432	0,928656		SD	0,32317
		4	2664,265	2791,342	0,954475		n	8
	**TG**	5	25113,54	13872,42	1,810321		SEM	0,114258
		6	28143,35	8732,873	3,22269		P=	0,001403
		7	20942,35	7982,852	2,623417			
		8	13564,56	8635,38	1,570812			

TG
1,736957

3,028395
2,538138
1,561057
1,810321
3,22269
2,623417
1,570812
2,261473
0,672512
8
0,237769
