# Supplementary material for: Cardiac effects of OPA1 protein promotion in a transgenic animal model
Source: PLoS One. 2024 Nov 21;19(11):e0310394. doi: 10.1371/journal.pone.0310394 (PMC11581344; doi:10.1371/journal.pone.0310394)
Supplement: S10 Fig — (PDF) [file pone.0310394.s010.pdf]

Supplementary information for Figure 12.

MFN1

			Area	*Percent*	*Optical density (%)*			
gel1	**WT**	1	18742,09	13,742	54,968		**WT**	**TG**
		2	12686,29	9,302	37,208		54,968	67,092
		3	7399,388	5,425	21,7		37,208	71,14
		4	17183,48	12,599	50,396		21,7	59,004
	**TG**	5	22876,5	16,773	67,092		50,396	38,488
		6	24256,21	17,785	71,14		38,632	50,532
		7	20118,14	14,751	59,004		56,012	49,648
		8	13122,7	9,622	38,488		74,668	38,636
							64,984	26,888
gel2	**WT**	1	13884,63	9,658	38,632			
		2	20131,12	14,003	56,012	mean	49,821	50,1785
		3	26835,48	18,667	74,668	SD	16,85023	15,19441
		4	23355,19	16,246	64,984	n	8	8
	**TG**	5	18161,58	12,633	50,532	SEM	5,957458	5,372036
		6	17843,65	12,412	49,648	P=	0,965083	
		7	13885,82	9,659	38,636			
		8	9663,045	6,722	26,888			

MFN2

			Area	*Percent*	*Optical density (%)*			
gel1	**WT**	1	16123,82	14,747	58,988			
		2	13173,34	12,049	48,196		**WT**	**TG**
		3	15343,46	14,034	56,136		58,988	66,3
		4	15224,63	13,925	55,7		48,196	58,06
	**TG**	5	18121,7	16,575	66,3		56,136	30,292
		6	15869,17	14,515	58,06		55,7	26,332
		7	8279,439	7,573	30,292		78,76	68,128
		8	7197,61	6,583	26,332		44,348	53,976
							72,844	20,632
gel2	**WT**	1	11896,02	19,69	78,76		54,492	6,82
		2	6698,368	11,087	44,348			
		3	11002,56	18,211	72,844	mean	58,683	41,3175
		4	8230,731	13,623	54,492	SD	11,66271	23,13801
	**TG**	5	10290,15	17,032	68,128	n	8	8
		6	8152,489	13,494	53,976	SEM	4,123392	8,180521
		7	3116,104	5,158	20,632	P=	0,078848	
		8	1029,82	1,705	6,82			

DRP1

			Area	*Percent*	*Optical density (%)*			
gel1	**WT**	1	11392,89	9,754	39,016			
		2	11379,36	9,743	38,972		**WT**	**TG**
		3	16835,72	14,414	57,656		39,016	69,132
		4	23259,33	19,914	79,656		38,972	47,112
	**TG**	5	20186,5	17,283	69,132		57,656	31,236
		6	13756,72	11,778	47,112		79,656	37,224
		7	9120,823	7,809	31,236		17,648	71,708
		8	10869,12	9,306	37,224		40,712	66,104
							51,008	45,636
gel2	**WT**	1	7434,045	4,412	17,648		53,316	53,872
		2	17150,72	10,178	40,712			
		3	21488,97	12,752	51,008	mean	47,248	52,753
		4	22461,09	13,329	53,316	SD	17,9869	15,08864
	**TG**	5	30210,28	17,927	71,708	n	8	8
		6	27848,74	16,526	66,104	SEM	6,35933	5,334639
		7	19225,67	11,409	45,636	P=	0,517969	
		8	22695,45	13,468	53,872			

Fis1

			Area	*Percent*	*Optical density (%)*			
gel1	**WT**	1	13841,46	15,219	60,876		**WT**	**TG**
		2	14721,05	16,187	64,748		60,876	67,512
		3	5876,66	6,462	25,848		64,748	48,756
		4	13221,39	14,538	58,152		25,848	45,096
	**TG**	5	15349,63	16,878	67,512		58,152	29,016
		6	11085,68	12,189	48,756		40,008	65,664
		7	10253,15	11,274	45,096		82,14	53,22
		8	6597,075	7,254	29,016		66,536	30,668
							41,5	20,264
gel2	**WT**	1	7318,075	10,002	40,008			
		2	15025,68	20,535	82,14	mean	54,976	45,0245
		3	12171,27	16,634	66,536	SD	17,99202	17,27192
		4	7591,075	10,375	41,5	n	8	8
	**TG**	5	12011,27	16,416	65,664	SEM	6,36114	6,106547
		6	9735,489	13,305	53,22	P=	0,27805	
		7	5610,246	7,667	30,668			
		8	3706,518	5,066	20,264			

PINK1

			Area	*Percent*	*Optical density (%)*			
gel1	**WT**	1	1826,083	0,090234	36,0935		**WT**	**TG**
		2	1792,669	0,088583	35,43306		36,0935	264,4286
		3	1498,598	0,074051	29,62059		35,43306	326,9769
		4	2207,912	0,109101	43,64055		29,62059	300,3329
	**TG**	5	13378,27	0,661071	264,4286		43,64055	230,9058
		6	16542,78	0,817442	326,9769		32,10792	296,6557
		7	15194,78	0,750832	300,3329		34,70628	327,5888
		8	11682,25	0,577264	230,9058		31,65149	313,9695
							45,81526	236,8635
gel2	**WT**	1	1624,44	0,08027	32,10792			
		2	1755,899	0,086766	34,70628	mean	36,13358	287,2152
		3	1601,348	0,079129	31,65149	SD	5,748704	38,56958
		4	2317,937	0,114538	45,81526	n	8	8
	**TG**	5	15008,74	0,741639	296,6557	SEM	2,032474	13,63641
		6	16573,74	0,818972	327,5888	P=	3,81E-11	
		7	15884,7	0,784924	313,9695	P=	<0,001	
		8	11983,66	0,592159	236,8635			

Parkin

			Area	*Percent*	*Optical density (%)*			
gel1	**WT**	1	7843,108	7,492	15,36444		**WT**	**TG**
		2	4948,004	4,726	18,53511		15,36444	142,6543
		3	10721,11	10,241	12,97271		18,53511	243,0738
		4	18751,64	17,912	15,24005		12,97271	100,392
	**TG**	5	1015,506	0,97	142,6543		15,24005	133,0598
		6	10570,64	10,097	243,0738		16,66705	175,2225
		7	44535,36	42,54	100,392		14,65897	272,5536
		8	6304,288	6,022	133,0598		13,82413	109,2474
							15,63916	129,9836
gel2	**WT**	1	11088,13	7,314	16,66705			
		2	2920,962	1,927	14,65897	mean	15,3627	163,2734
		3	1698,406	1,12	13,82413	SD	1,708443	62,98769
		4	28449,28	18,765	15,63916	n	8	8
	**TG**	5	39358,05	25,96	175,2225	SEM	0,604026	22,26951
		6	9237,338	6,093	272,5536	P=	1,12E-05	
		7	55279,17	36,462	109,2474	P=	<0,001	
		8	3576,276	2,359	129,9836			
