# Supplementary material for: Cardiac effects of OPA1 protein promotion in a transgenic animal model
Source: PLoS One. 2024 Nov 21;19(11):e0310394. doi: 10.1371/journal.pone.0310394 (PMC11581344; doi:10.1371/journal.pone.0310394)

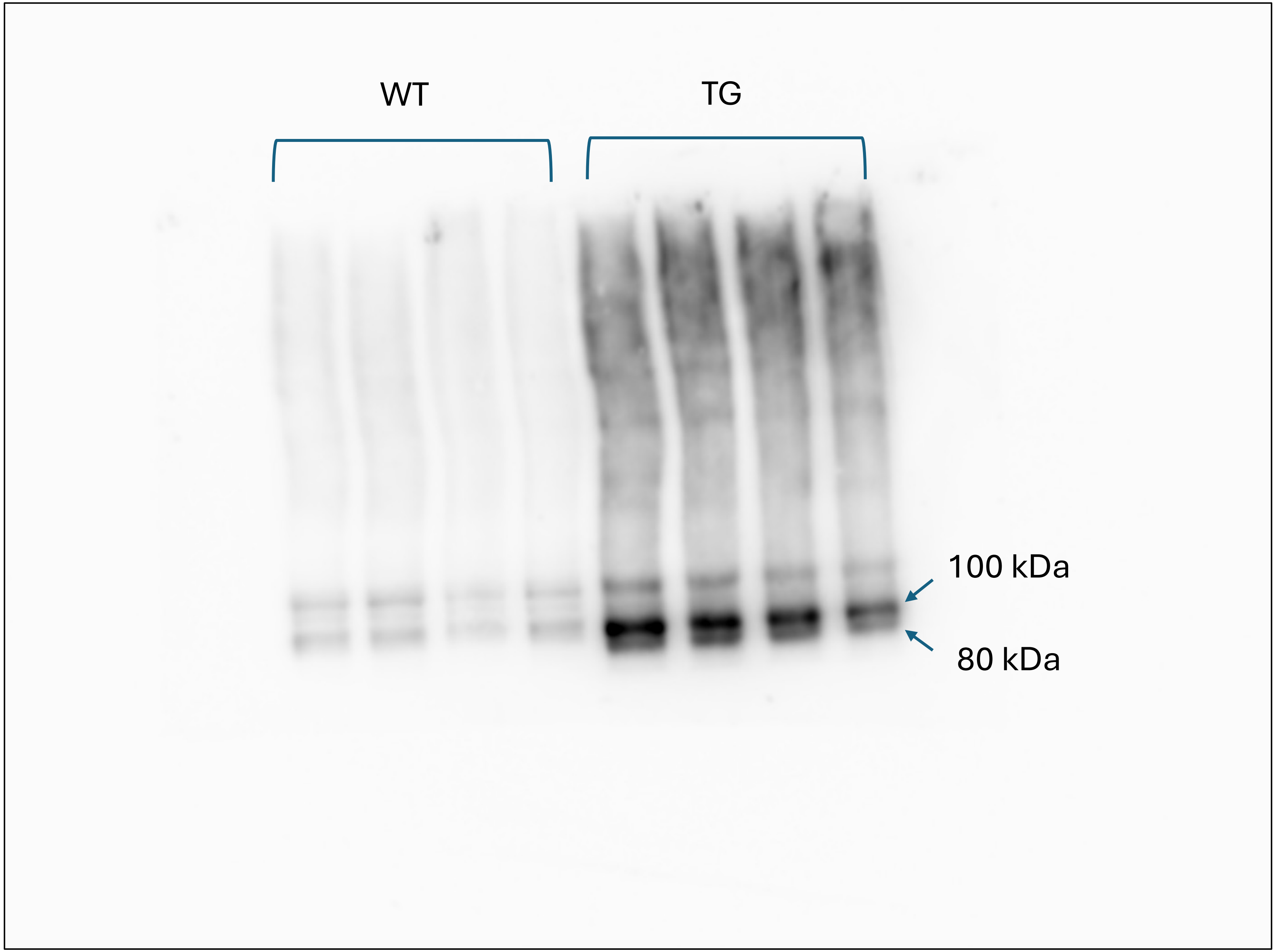

Fig 11.

OPA1

Fig 11.

FLAG

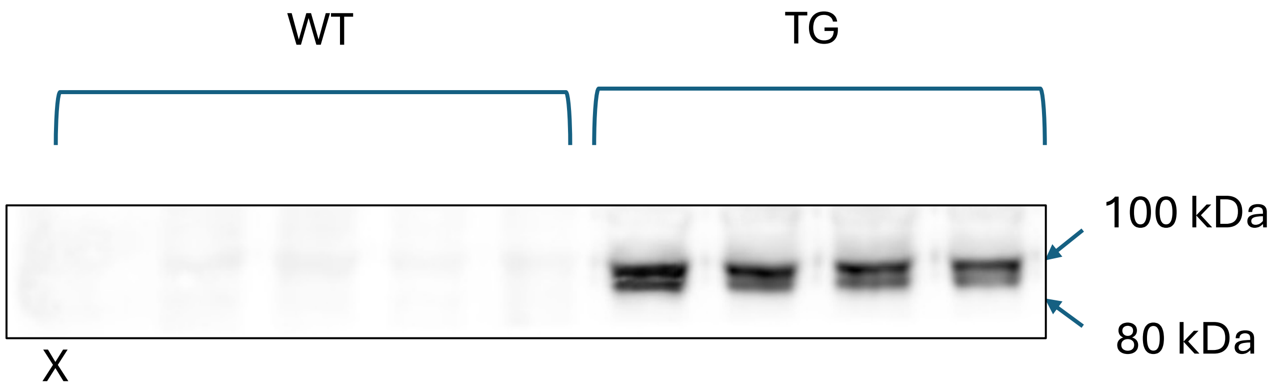

Fig 11.

YME1L1

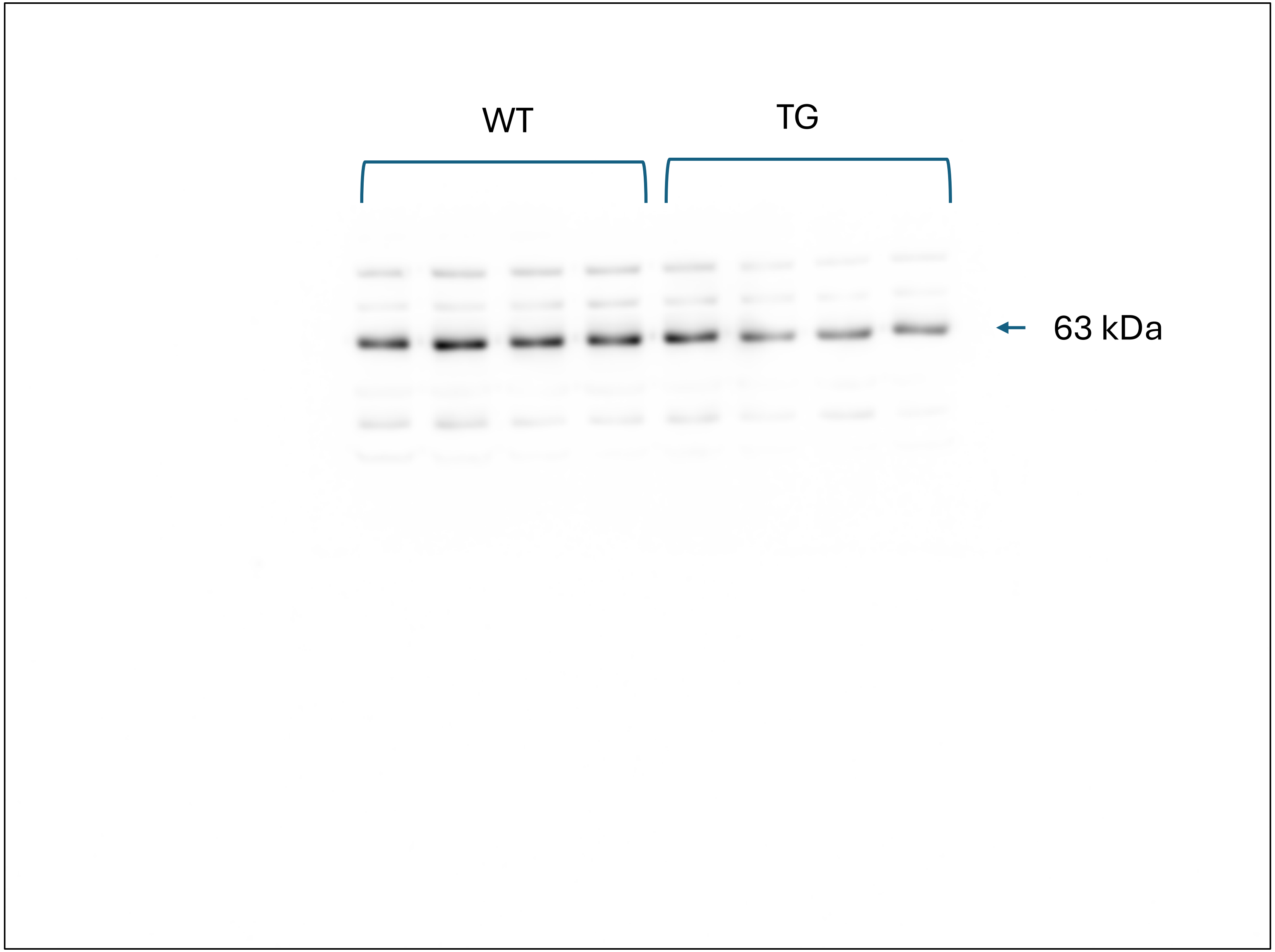

Fig 11.

OMA1

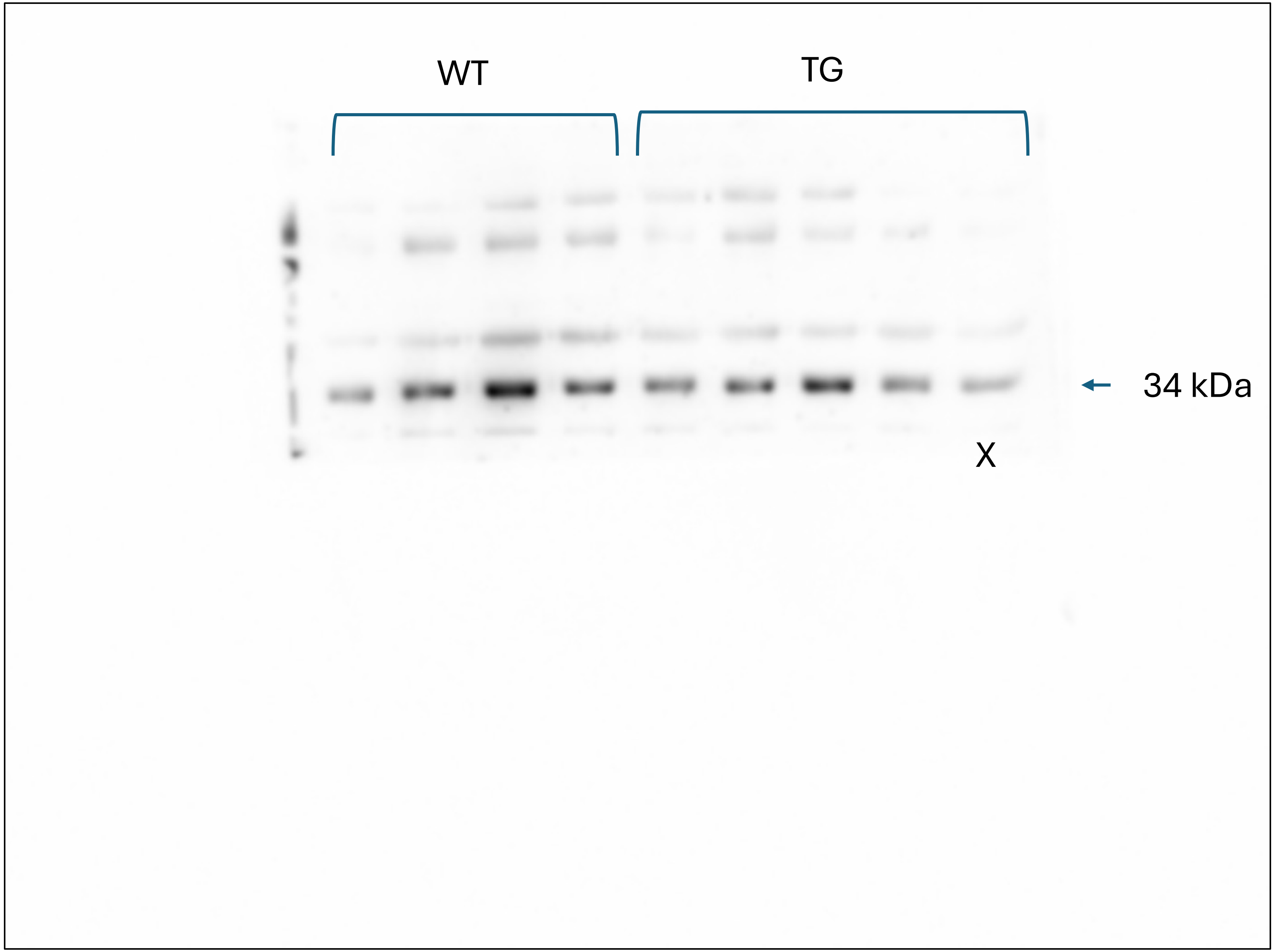

Fig 11.

VDAC

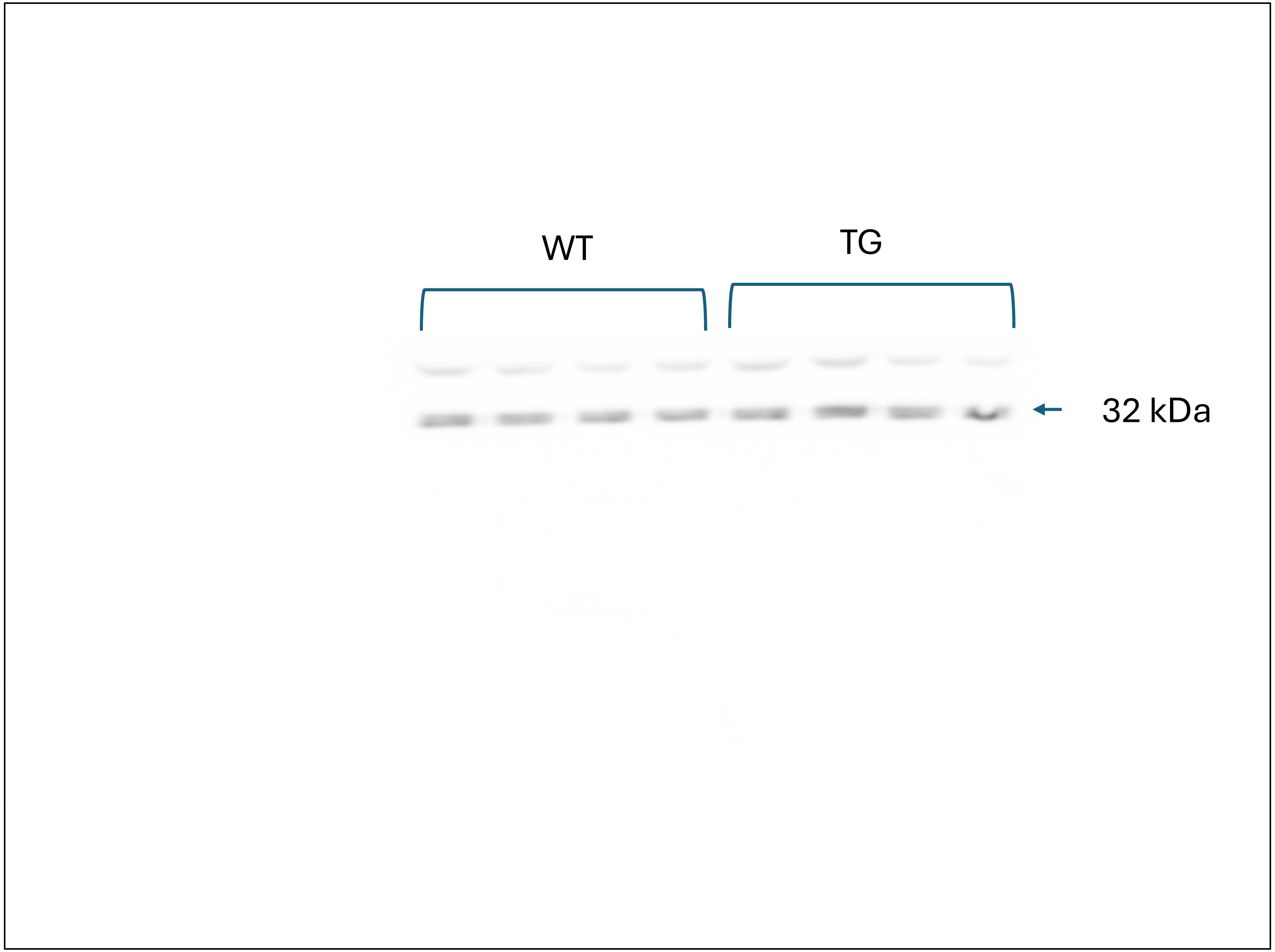

Fig 11.

BNIP3

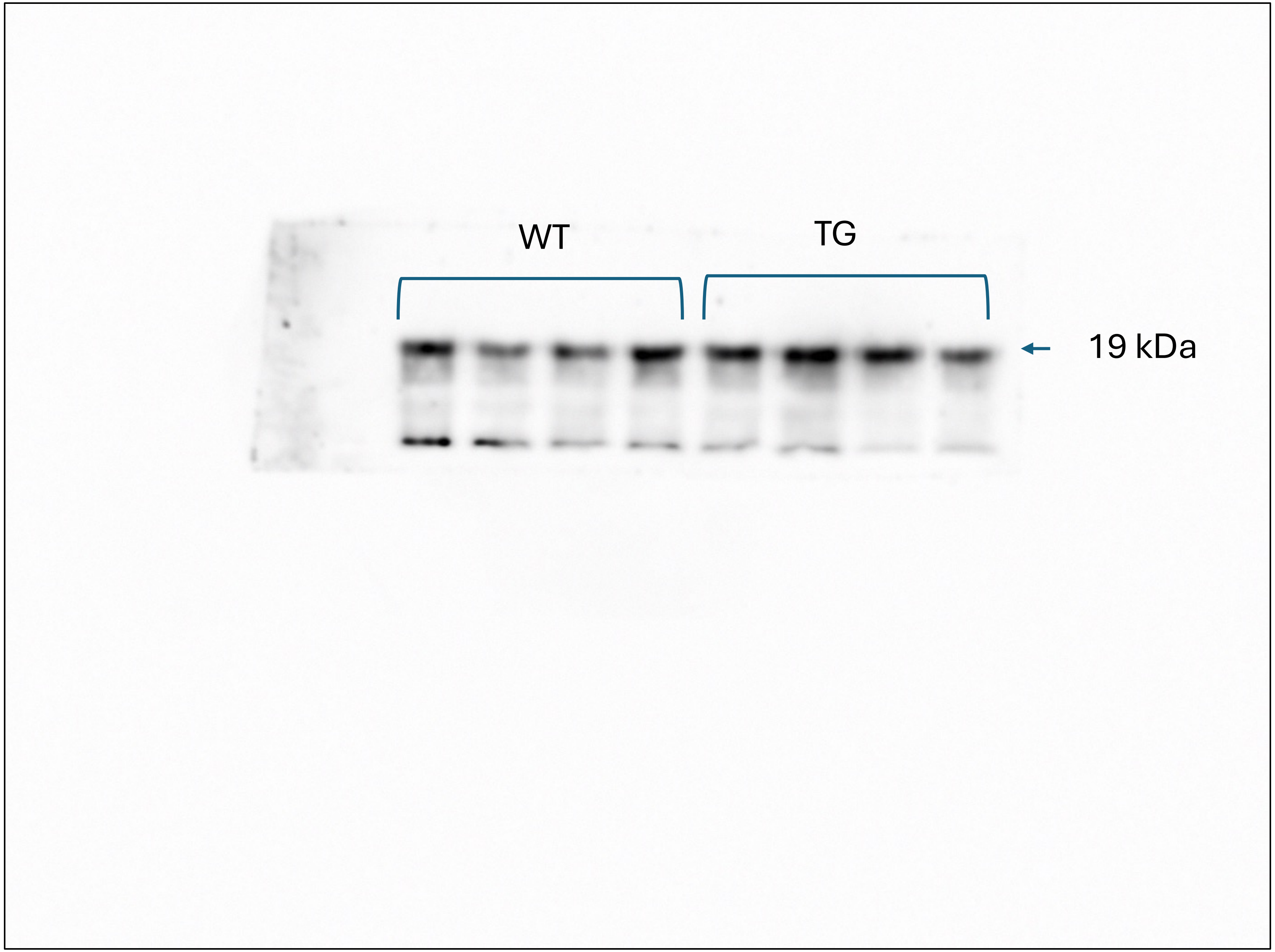

Fig 12.

Mfn1

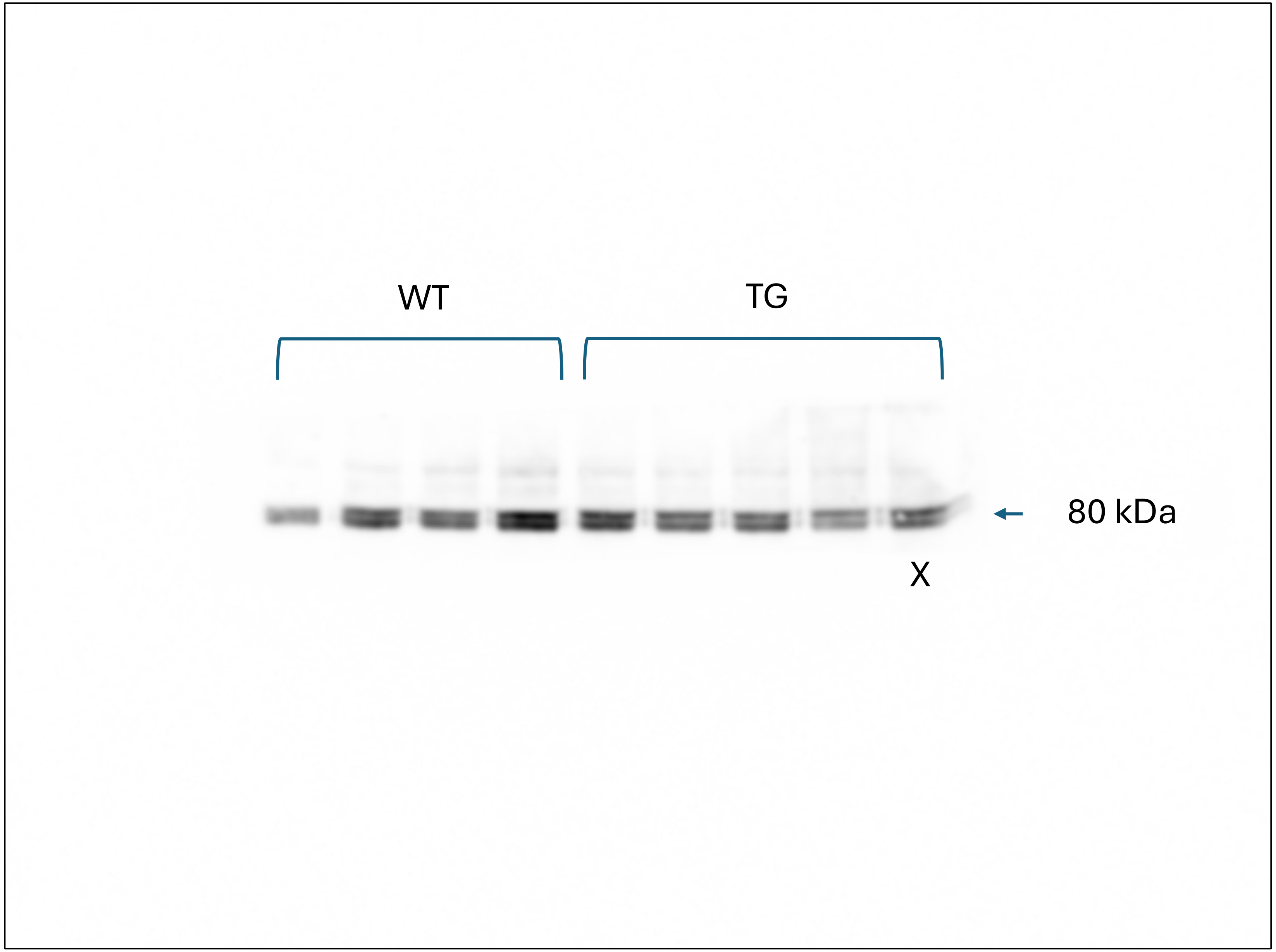

Fig 12.

Mfn2

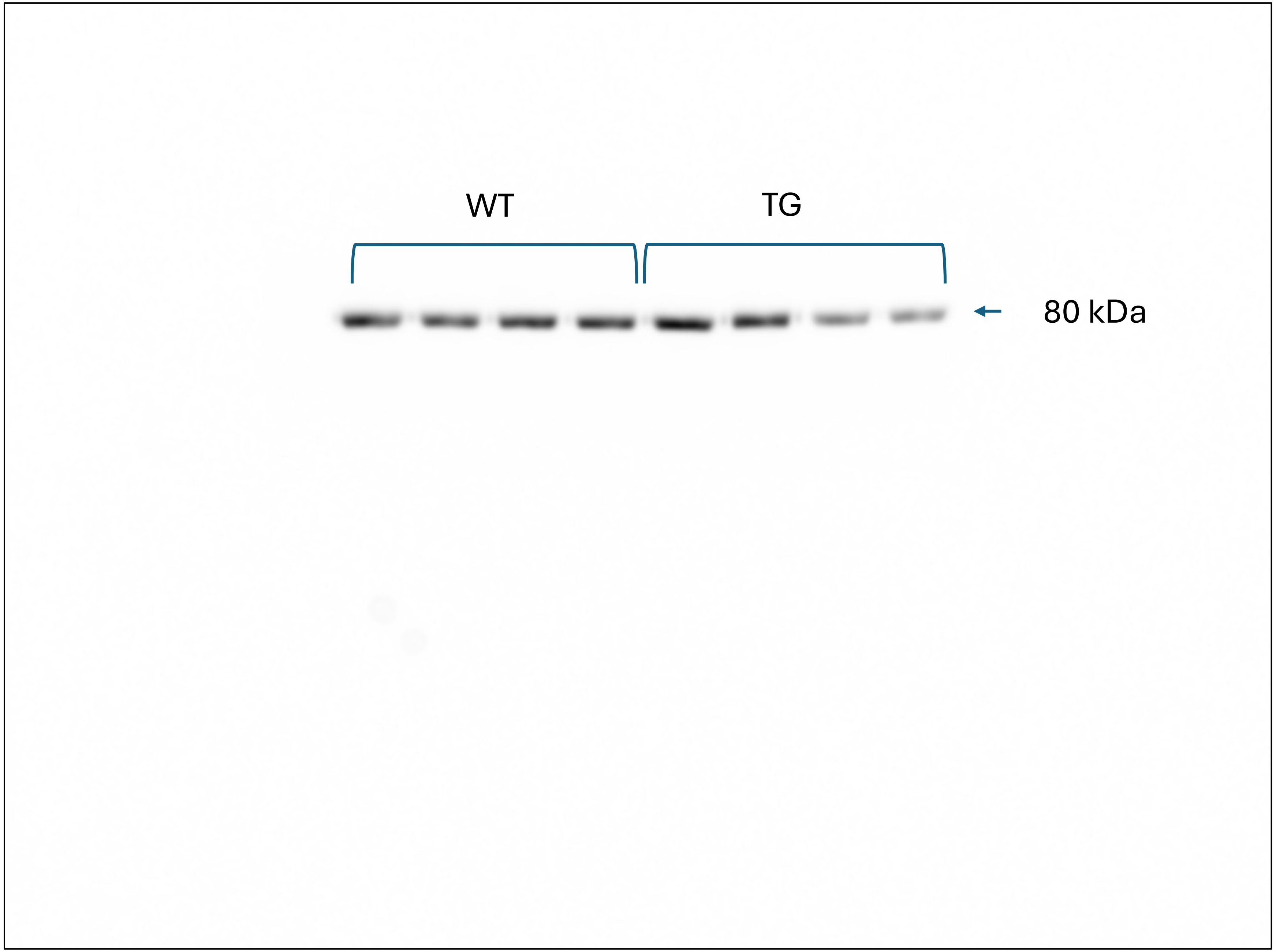

Fig 12.

Drp1

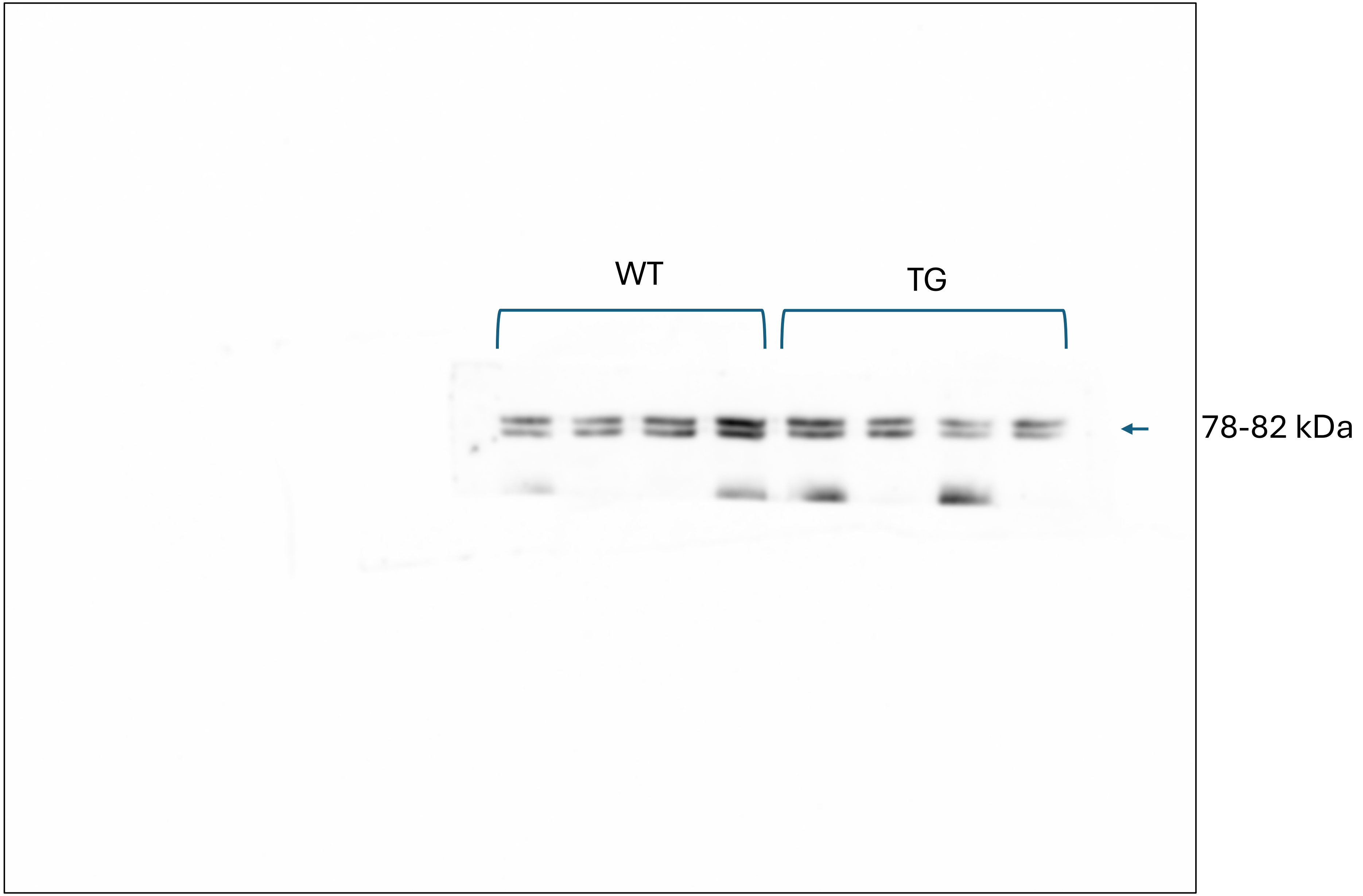

Fig 12.

Fis1

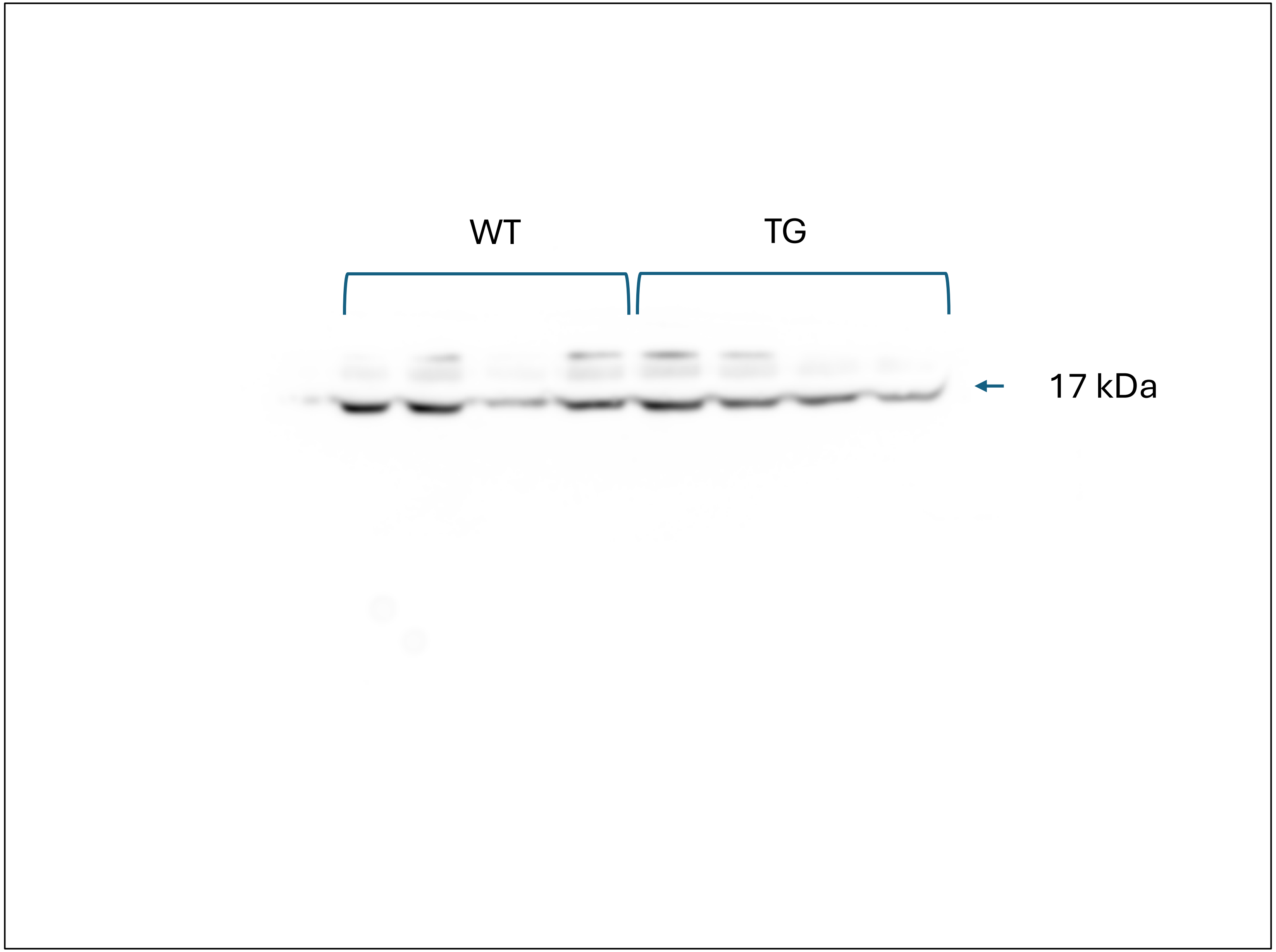

Fig 12.

PINK1

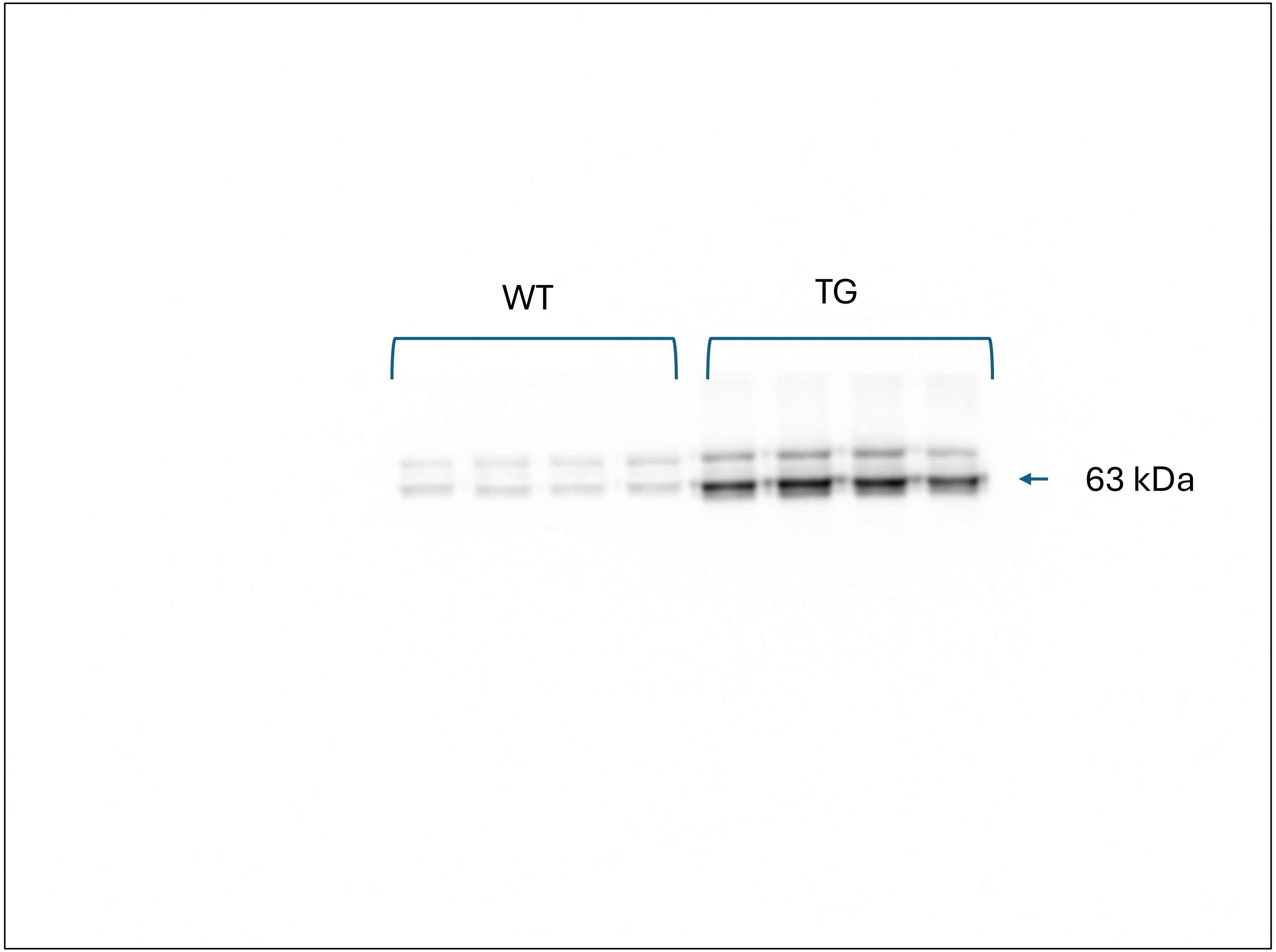

Fig 12.

Parkin

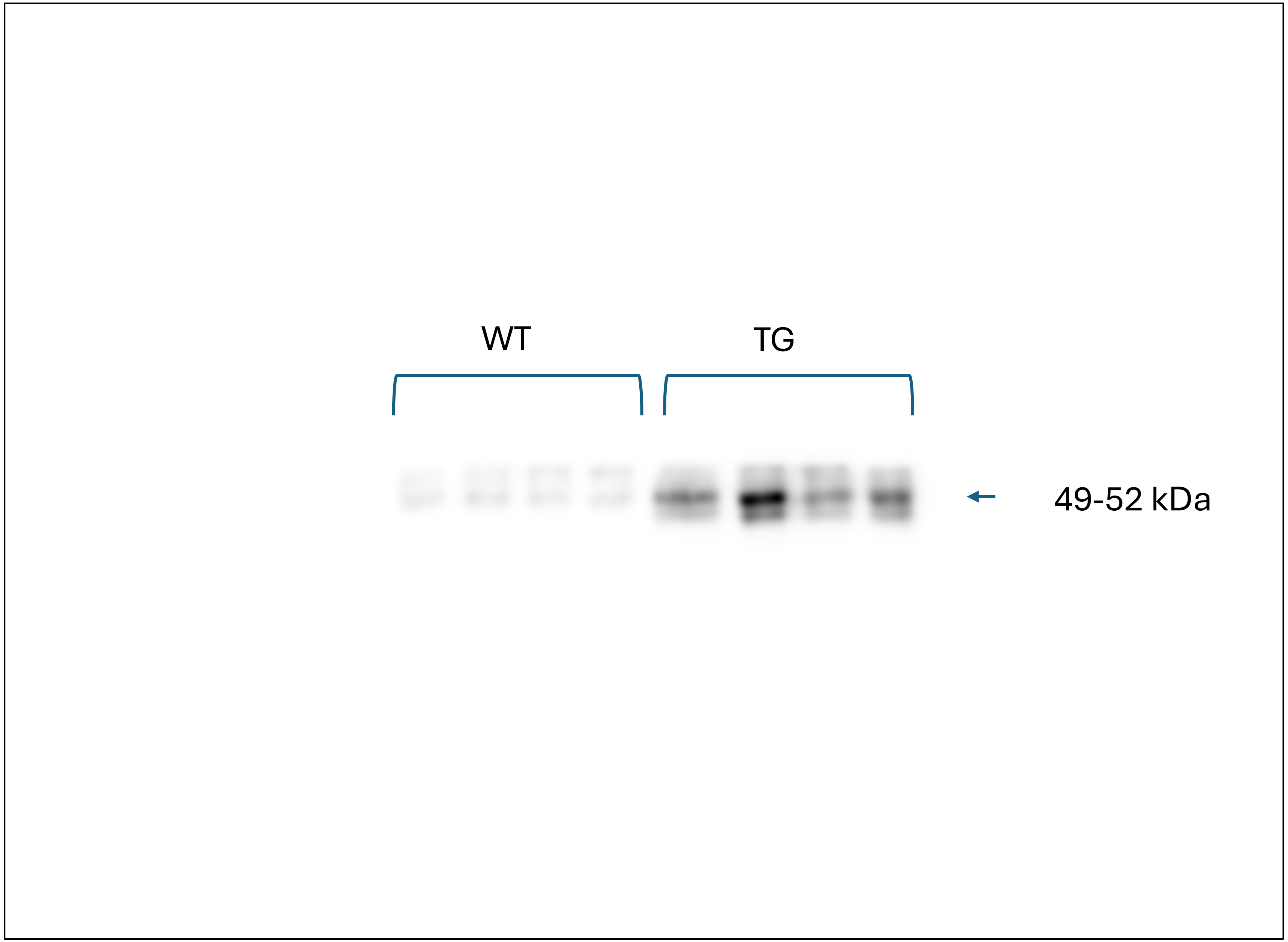

Supplement: S11 Fig — (PDF) [file pone.0310394.s011.pdf]
